# Angiotensin receptor blockers and β-blockers in Marfan syndrome: an individual patient data meta-analysis of randomised trials

**DOI:** 10.1016/S0140-6736(22)01534-3

**Published:** 2022-08-29

**Authors:** Alex Pitcher, Enti Spata, Jonathan Emberson, Kelly Davies, Heather Halls, Lisa Holland, Kate Wilson, Christina Reith, Anne H Child, Tim Clayton, Matthew Dodd, Marcus Flather, Xu Yu Jin, George Sandor, Maarten Groenink, Barbara Mulder, Julie De Backer, Arturo Evangelista, Alberto Forteza, Gisela Teixido-Turà, Catherine Boileau, Guillaume Jondeau, Olivier Milleron, Ronald V. Lacro, Lynn A Sleeper, Hsin-Hui Chiu, Mei-Hwan Wu, Stefan Neubauer, Hugh Watkins, Hal Dietz, Colin Baigent

**Affiliations:** The Heart Centre, John Radcliffe Hospital, Oxford University Hospitals NHS Foundation Trust, Oxford, UK; MRC Population Health Research Unit, Nuffield Department of Population Health, University of Oxford, Oxford, UK; Clinical Trial Service Unit & Epidemiological Studies Unit, Nuffield Department of Population Health, University of Oxford, Oxford, UK; MRC Population Health Research Unit, Nuffield Department of Population Health, University of Oxford, Oxford, UK; Clinical Trial Service Unit & Epidemiological Studies Unit, Nuffield Department of Population Health, University of Oxford, Oxford, UK; MRC Population Health Research Unit, Nuffield Department of Population Health, University of Oxford, Oxford, UK; Clinical Trial Service Unit & Epidemiological Studies Unit, Nuffield Department of Population Health, University of Oxford, Oxford, UK; MRC Population Health Research Unit, Nuffield Department of Population Health, University of Oxford, Oxford, UK; Clinical Trial Service Unit & Epidemiological Studies Unit, Nuffield Department of Population Health, University of Oxford, Oxford, UK; MRC Population Health Research Unit, Nuffield Department of Population Health, University of Oxford, Oxford, UK; Clinical Trial Service Unit & Epidemiological Studies Unit, Nuffield Department of Population Health, University of Oxford, Oxford, UK; Clinical Trial Service Unit & Epidemiological Studies Unit, Nuffield Department of Population Health, University of Oxford, Oxford, UK; Clinical Trial Service Unit & Epidemiological Studies Unit, Nuffield Department of Population Health, University of Oxford, Oxford, UK; Royal Brompton and Harefield Hospitals Unit, Guy’s and St Thomas’ NHS Trust and Department of Surgery and Oncology, Imperial College, London; Clinical Trials Unit, Department of Medical Statistics, London School of Hygiene & Tropical Medicine, London, UK; Clinical Trials Unit, Department of Medical Statistics, London School of Hygiene & Tropical Medicine, London, UK; Faculty of Medicine and Health Sciences, University of East Anglia, Norwich, UK; The Heart Centre, John Radcliffe Hospital, Oxford University Hospitals NHS Foundation Trust, Oxford, UK; Children’s Heart Centre, British Columbia’s Children’s Hospital, Vancouver, British Columbia, Canada; Department of Paediatrics, Faculty of Medicine, University of British Columbia, Vancouver, Canada; Academic Medical Center, University of Amsterdam, Amsterdam, The Netherlands; Center for Medical Genetics and Department of Cardiology, Ghent University Hospital, Ghent, Belgium; Servei de Cardiologia. Hospital Universitari Vall d’Hebron. Barcelona; Hospital Puerta de Hierro, Majadahonda, Spain; Department of Cardiology, Hospital Universitari Vall d’Hebron, CIBER-CV, Vall d’Hebron institut de Recerca, Universitat Autònoma de Barcelona, Barcelona, Spain; Université Paris Cité and Université Sorbonne Paris Nord, Inserm U1148, LVTS, F-75018 Paris, France; Service de Cardiologie, AP-HP, Hôpital Bichat-Claude Bernard, F-75018, France; CRMR Syndrome de Marfan et apparentés, AP-HP Hôpital Bichat-Claude Bernard, F-75018, France; Université Paris Cité and Université Sorbonne Paris Nord, Inserm U1148, LVTS, F-75018 Paris, France; Service de Cardiologie, AP-HP, Hôpital Bichat-Claude Bernard, F-75018, France; CRMR Syndrome de Marfan et apparentés, AP-HP Hôpital Bichat-Claude Bernard, F-75018, France; Université Paris Cité and Université Sorbonne Paris Nord, Inserm U1148, LVTS, F-75018 Paris, France; Service de Cardiologie, AP-HP, Hôpital Bichat-Claude Bernard, F-75018, France; CRMR Syndrome de Marfan et apparentés, AP-HP Hôpital Bichat-Claude Bernard, F-75018, France; Department of Cardiology, Boston Children’s Hospital, Boston, USA; Department of Pediatrics, Harvard Medical School, Boston, MA USA; Department of Cardiology, Boston Children’s Hospital, Boston, USA; Department of Pediatrics, Harvard Medical School, Boston, MA USA; Department of Pediatrics, Taipei Tzu Chi Hospital, Buddhist Tzu Chi Medical Foundation, New Taipei City, Taiwan; Department of Pediatrics and Adult Congenital Heart Center, National Taiwan University Hospital, Taipei, Taiwan; Oxford Centre for Clinical Magnetic Resonance Research, Division of Cardiovascular Medicine, Radcliffe Department of Medicine, University of Oxford, Oxford, UK; Radcliffe Department of Medicine, University of Oxford, Oxford, UK; Howard Hughes Medical Institute and Department of Genetic Medicine, Johns Hopkins University School of Medicine, Baltimore, Maryland, USA; MRC Population Health Research Unit, Nuffield Department of Population Health, University of Oxford, Oxford, UK

## Abstract

**Background:**

Angiotensin Receptor Blockers (ARBs) and β-blockers are widely used in the treatment of Marfan syndrome to try to reduce the rate of progressive aortic root enlargement characteristic of this condition, but their separate and joint effects are uncertain. We aimed to determine these effects in a collaborative individual patient data meta-analysis of randomised trials of these treatments.

**Methods:**

Meta-analysis of individual data from 1,442 patients with Marfan syndrome and no prior aortic surgery in seven randomised trials was performed. Estimates were obtained of the effects of: (i) ARB versus control (placebo or open control), (ii) ARB versus β-blocker, and (iii) indirectly, β-blocker versus control. The primary endpoint was the annual rate of change of body surface area (BSA)-adjusted aortic root dimension Z score, measured at the sinuses of Valsalva.

**Findings:**

Four trials involving 676 eligible participants compared ARB with control. During a median follow-up of 3 years, allocation to ARB approximately halved the annual rate of change in the aortic root Z score (annual increase +0.07 (SE 0.02) ARB versus +0.13 (SE 0.02) control, absolute difference -0.07 [95% CI: -0.12 to -0.01, p=0.01]). The effect of an ARB on change in aortic root Z score was larger in those with a known *FBN1* pathogenic variant than in those without such a variant (p for heterogeneity = 0.005), but there was no evidence that the effect was modified by any other patient characteristic. In particular, the effect of an ARB was similar whether or not a patient was also prescribed a β-blocker at baseline. Few patients experienced the composite outcome of aortic dissection, aortic root surgery or death during study follow-up (30 [8%] ARB vs 27 [8%] control, p=0.86). Three trials involving 766 eligible participants compared ARB vs β-blocker. During a median followup of 3 years, the annual change in the aortic root Z score was similar in the two groups (annual increase -0.08 (SE 0.03) ARB vs -0.11 (SE 0.02) β-blocker, absolute difference 0.03 [95% CI -0.05 to 0.10], p=0.48). Thus, indirectly, the difference in the annual change in the aortic root Z score between β-blocker and control was -0.09 (95% CI -0.18 to 0.00, p=0.04).

**Interpretation:**

In patients with Marfan syndrome and no prior aortic surgery, ARBs reduced the rate of increase of the aortic root Z score by about one half, including among those taking a β-blocker. The effects of β-blockers were similar to those of ARBs. Assuming additivity, combination therapy with both ARBs and β-blockers from the time of diagnosis would provide even greater reductions in the rate of aortic enlargement than either treatment alone which, if maintained over a number of years, would be expected to lead to a delay in the need for aortic surgery.

## Introduction

Marfan syndrome is a genetic disorder, usually caused by pathogenic variants in the Fibrillin 1 (*FBN1*) gene that causes progressive enlargement of the aortic root. If unchecked, aortic enlargement in Marfan syndrome can lead to life-threatening aortic dissection, sometimes in early adulthood.^1–7^ Initial treatment is aimed at slowing aortic root growth, and β-blockers are widely used for this purpose, but their use is based mainly on the results of observational studies^8,9^ and one small randomised trial.^10^

The discovery that Transforming growth factor β (TGF β) dysregulation is implicated in the pathogenesis of some aortic aneurysms led to the hypothesis that angiotensin receptor blockade (which attenuates TGFβ activity) might slow aortic root growth in Marfan syndrome.^11,12^ Favourable results in animal models^11^ and in small observational studies in humans^12–14^ led to several randomised trials in patients with Marfan syndrome in the last decade.^15–24^ However, more precise estimates of treatment effect and more powerful subgroup analyses are attainable by combining these trials. In 2012 we established a collaborative group (the Marfan Treatment Trialists’ [MTT] Collaboration) in order to conduct a meta-analysis of individual-patient-data from all relevant Marfan syndrome trials. A protocol was subsequently agreed for the rationale, design and conduct of the meta-analysis.^25^

Since 2012 most of the participating trials have reported their results,^15–18,20–24^ and several meta-analyses of published data have been reported.^26–28^ Two of these meta-analyses ^26,28^ did not include the recent UK-based AIMS trial ^23^, but one that did include this trial^27^ concluded that ARBs are effective when used alone or when added to a β-blocker. Since this meta-analysis included only published data, it could not avoid any biases that might arise from selective reporting of findings in publications, nor could it harmonise definitions of aortic root size for all trials or explore treatment effects in more detail (for example, among particular prognostic subgroups, such as those with a confirmed *FBN1* pathogenic variant). The present report describes a meta-analysis in which the availability of individual-patient-data allowed these limitations to be overcome, allowing not only a more complete assessment of ARBs in Marfan syndrome, but also − by pre-specifying the use of indirect comparisons of trials of an ARB vs control and of an ARB vs β-blocker − an assessment of the effects of β-blocker therapy given alone and the effects of combined ARB and β-blocker therapy.

## Methods

Relevant trials were identified by systematically searching Medline, Embase and CENTRAL up to 2^nd^ November 2021. Trials were eligible if they involved a randomised comparison of an ARB versus control or an ARB versus β-blocker in patients with Marfan syndrome. A description of the search terms is given in Webtable 1. The review of search results was conducted independently by two authors and adjudicated by a third. Relevant trials were also sought through enquiry amongst collaborating trialists.^25^

The primary aims of this meta-analysis were to estimate the effects of (i) ARB and (ii) β-blockers on the change in aortic root size in patients with Marfan syndrome and no prior aortic root surgery. (Thus, the small number of patients with prior aortic root surgery in the identified trials were excluded from analyses.) The primary comparisons involved only the unconfounded trials (ie, where there were no protocol-mandated differences between randomised groups other than those created by the randomised allocations), but a sensitivity analysis included one trial^22^ in which there were different dosing strategies for β-blockers in the ARB and control arms of the study. The pre-specified primary outcome was the annual rate of change of body surface area (BSA)-adjusted aortic root dimension Z score, measured at the sinuses of Valsalva. The secondary outcome was the annual rate of change of the absolute aortic root dimension measured at the sinuses of Valsalva. Other secondary aims were to assess those effects across different subgroups defined based on patients’ characteristics at baseline, to assess effects on cardiovascular outcomes, including aortic dissection, aortic root surgery and death, as well as the composite of these three outcomes; and to assess effects on a range of other outcomes and measures where these were sufficiently complete to permit meaningful analyses (see Webtables 2 and 3). Z scores were calculated using the method used by each trial (as reported in their main analysis) except where values were provided directly by the trialist. Secondary analyses using the methods of Campens et al^29^ and Pettersen et al^30^ were performed to explore the impact of using an alternative method to estimate aortic root Z score. A two-stage meta-analysis approach was used. For each patient, a linear slope of the annual rate of change (from baseline) of the BSA-adjusted Z score was calculated. The difference in mean slopes between treatment arms (and its standard error) was then calculated for each trial and standard inverse-variance-weighted methods were used to estimate the overall inverse-variance-weighted average difference in slopes across all trials. (A ‘random effects’ meta-analysis, which assumes that the underlying set of trials are representative of an underlying population of possible trial designs, was also done as a sensitivity analysis.) The small percentage of patients with missing data on rate of change in aortic root Z score were excluded. Pre-specified subgroup analyses according to baseline participant characteristics were performed. To allow for multiple subdivisions of the data, only summary effect estimates are presented with 95% confidence intervals (CIs); all other effect estimates (such as results from individual trials or in particular patient subgroups) are presented with 99% CIs.

An indirect assessment of the mean effect of β-blocker compared to control was made using indirect comparisons of trial results^31^ as follows: if d_1_ (with variance v_1_) is the difference in mean annual rate of change in aortic root Z scores estimated from the three trials that compared ARB versus β-blockers and d2 (with variance v2) is the difference in mean annual rate of change in aortic root Z scores estimated from the four trials that compared ARB versus control, then an indirect estimate of the effect of β-blockers is provided by d_2_ - d_1_ (which has variance equal to v_1_ + v_2_). This indirect analysis makes the assumption that the effects of ARBs and β-blockers are additive (that is, the effect of an ARB is the same whether a β-blocker is given or not and the effect of a β-blocker is the same irrespective of whether an ARB is given or not).

For the primary comparisons, a two-sided p-value less than 0.05 was considered statistically significant. Analyses were performed using SAS version 9.4 (SAS Institute, Cary) and R version 3.5.0 (www.R-project.org).

The funders had no role in the study design, data collection, analysis, interpretation, writing of the manuscript or the decision to submit.

## Results

Ten trials (1836 patients, Table 1, Webtable 1, Webfigure 1) were identified as being potentially eligible for the study: Bhatt et al, 2015 (NCT00723801); COMPARE, 2013 (NTR1423); Gambarin et al 2019 (NCT00683124); Ghent Marfan, 2017 (NCT00782327); Hsin-Hui Chiu et al, 2013 (NCT00651235); LOAT, 2016 (NCT001145612); Marfan-Sartan, 2015 (NCT00763893); Pediatric Heart Network (PHN), 2014 (NCT00429364); Sandor et al, 2015 (NCT00593710); UK AIMS, 2019 (ISRCTN90011794).^15–24^ Three (involving 324 patients) were not included in the primary analyses: one (262 patients) was published as an abstract only and was unable to contribute data;^32^ another (34 patients) was published but unable to contribute data;^16^ and a third (28 patients) contributed data but was found to be confounded owing to protocol-mandated adjustment in β-blocker doses in the control arm.^22^ (This third trial contributed only to a sensitivity analysis.) Of the seven remaining trials, 70 (4.6%) of the 1512 randomised patients were excluded because they had had prior aortic root surgery. The main analyses therefore include individual data from 1442 participants in seven trials.^15,17,18,20,21,23,24^

**Figure 1:**
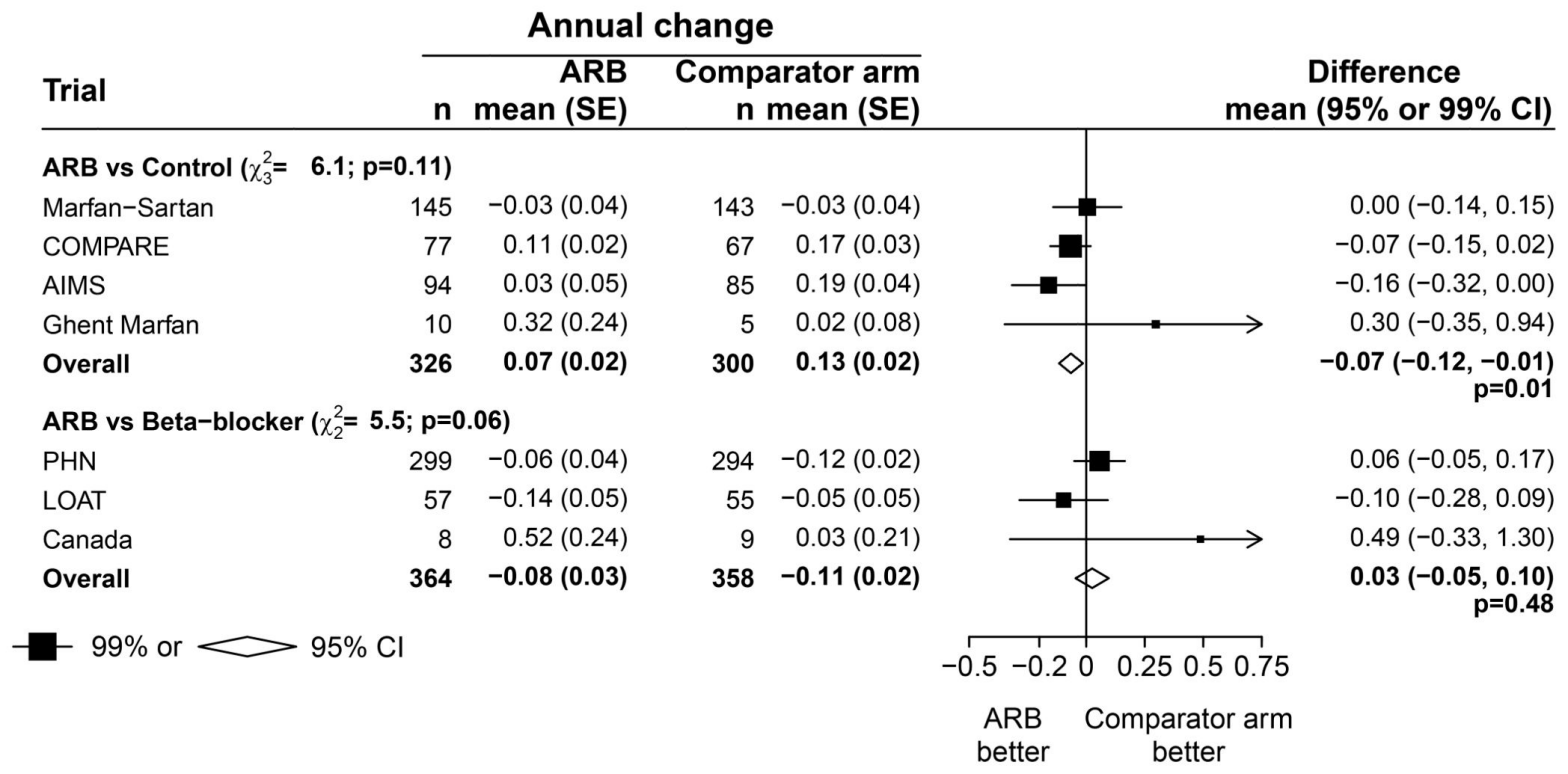
Annual rate of change of BSA-adjusted aortic root dimension Z score at the sinuses of Valsalva Indirect effect of β−blocker vs control: -0.09 (95% CI: -0.18 to 0.00), p-value=0.04 (β–blocker minus control).

Data were available from four trials of ARB versus control, including 676 patients (353 ARB versus 323 control).^15,18,20,23^ The mean age of participants in these trials was 29 years (SD 14), 54% were female and 75% were receiving β-blocker at baseline (all allowed patients to remain on their β-blocker). Overall, 83% of genotyped individuals had an *FBN1* pathogenic variant (Table 2). The mean Z score at the sinuses of Valsalva at baseline was 3.76 (SD 2.14) in patients allocated an ARB and 3.64 (SD 1.94) in patients allocated control. The mean annual change of the Z score during follow up was 0.07 (SE 0.02) in the ARB arm and 0.13 (SE 0.02) in the control arm, corresponding to a mean difference of -0.07 (95% CI: -0.12 to - 0.01, p=0.012; Figure 1, **upper panel**), which represented an approximate halving in the annual rate of change in the aortic root Z score.

**Figure 2:**
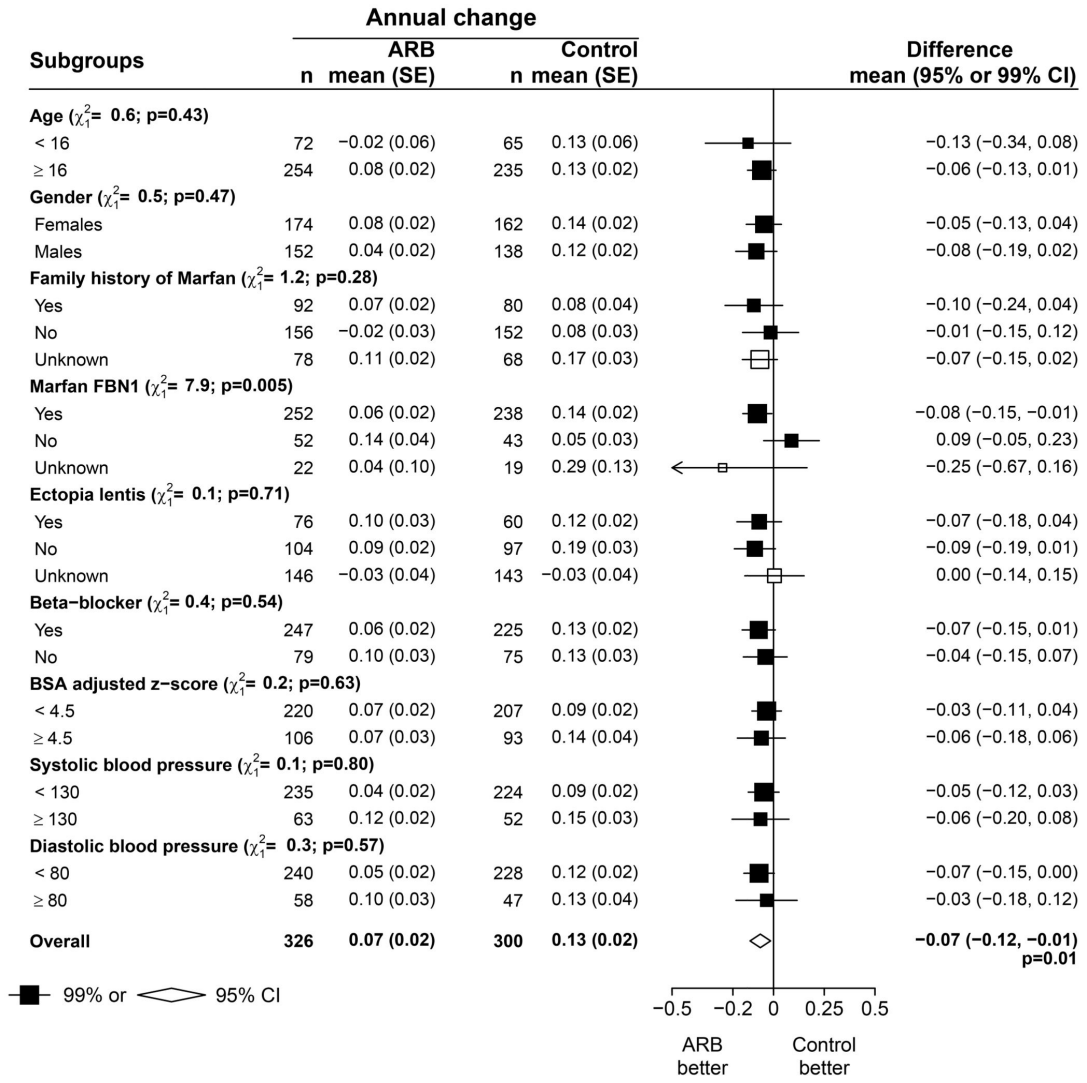
ARB vs control: annual rate of change of BSA-adjusted aortic root dimension Z score at the sinuses of Valsalva, by subgroups

Although there was no evidence of heterogeneity between the overall results of the four contributing trials (heterogeneity p=0.11, Figure 1), within these trials there was significant heterogeneity of treatment effects with an ARB between the 490 participants with a documented pathogenic variant in *FBN1*, compared with the 95 participants who definitely did not have a pathogenic variant in *FBN1* (Figure 2 and Webfigure 2, p heterogeneity=0.005). There was no significant heterogeneity of the effects of an ARB in any of the other pre-specified subgroups however. In particular, the mean annual change in aortic root Z score was similar irrespective of whether patients were taking a β-blocker at baseline (heterogeneity p=0.54).

Secondary analyses were conducted to assess the sensitivity of the findings to different methods for measuring aortic root size. For the secondary outcome of absolute aortic dimension, the mean annual change was 0.38 mm (SE 0.04) in patients allocated ARB and 0.52 mm (SE 0.04) in patients allocated control, resulting in a mean difference of -0.14 mm (95% CI: -0.26 to -0.02, p=0.025; Webfigure 3). Findings were similar when the analysis included one confounded trial^22^ in which there were different dosing strategies for β-blockers in the ARB and control arms during the study (mean difference -0.08 [95% CI -0.13 to -0.03]; p=0.001; Webfigure 4), and were also similar when the Z scores were calculated using the method described by Campens et al^29^ (mean difference =-0.04, 95%CI -0.07 to -0.01; p=0.007; Webfigure 5) or by Pettersen et al^30^ (mean difference =-0.04, 95%CI -0.07 to -0.01);p=0.017; Webfigure 6).

In the trials of ARB vs. control, there was no significant difference in the proportion experiencing the composite outcome of aortic dissection, aortic root surgery or death during study follow-up (30 [8%] ARB vs. 27 [8%] control, p=0.86). Nor was there any evidence of difference in this composite outcome in the trials of ARB vs β-blocker (21 [5%] ARB vs. 14 [4%] β-blocker, p=0.23; Webtable 2).

**Figure 3:**
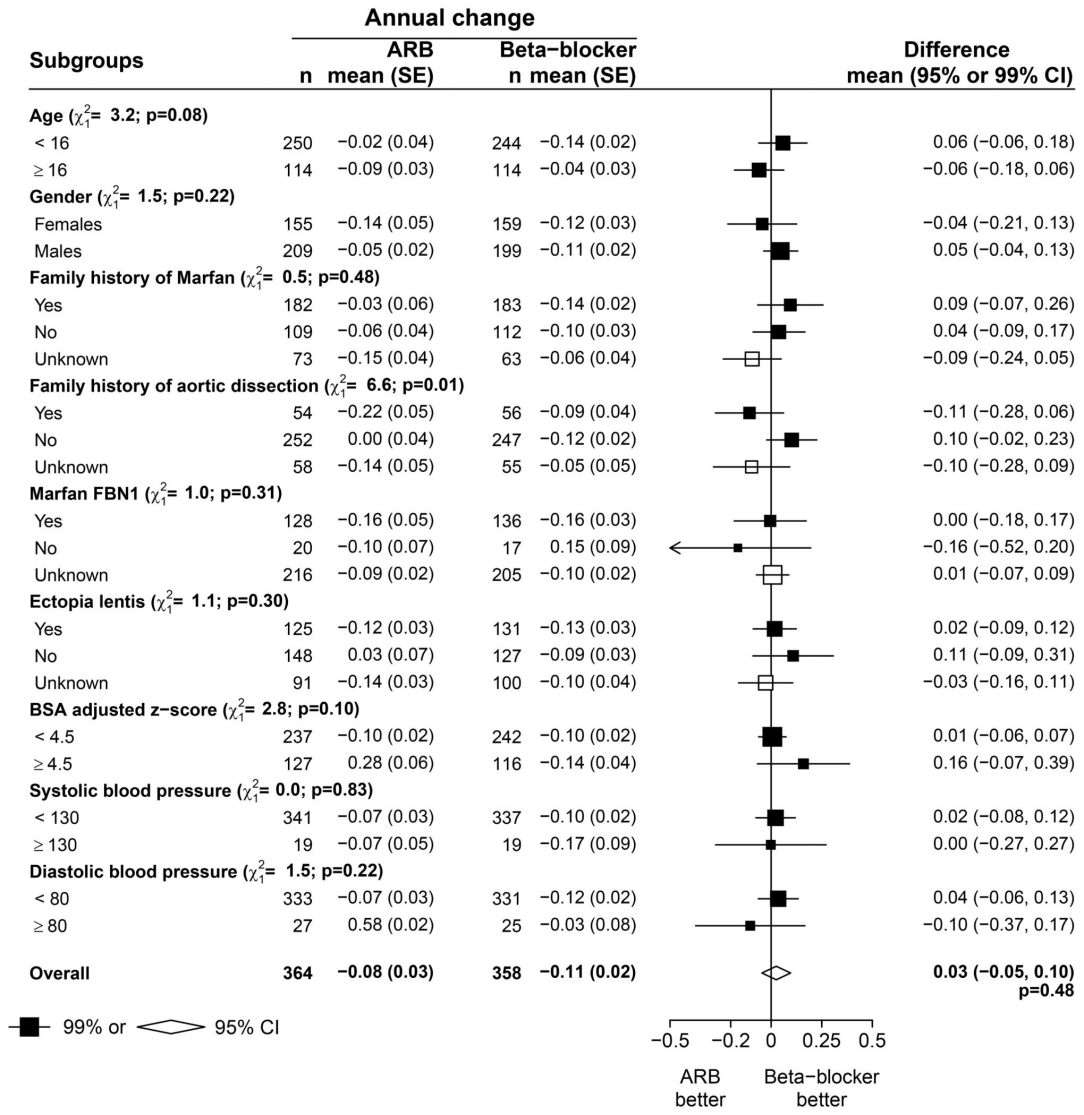
ARB vs β-blocker: annual rate of change of BSA-adjusted aortic root dimension Z score at the sinuses of Valsalva, by subgroups

Individual participant data were available from three trials of ARB versus β-blocker, including 766 patients (384 ARB versus 382 β-blocker).^17,21,24^. The mean age of participants was 14 years (SD 10); 44% were female, and 86% of genotyped individuals had a pathogenic variant in *FBN1* (Table 2). The baseline mean Z score was 4.18 (SD 1.71) in patients allocated an ARB and 4.03 (SD 1.50) in patients allocated β-blocker. The mean annual change of the Z score during follow-up was -0.08 (SE 0.03) in the ARB arm and -0.11 (SE 0.02) in the β-blocker arm, and the mean difference in the change of Z scores between ARBs and β-blockers was not significant (0.03, 95% CI: -0.05 to 0.10, p=0.484; Figure 1, lower panel). There were no significant differences in aortic Z score when using other methods^29,30^ for calculating it (Webfigures 5 and 6). Similarly, there were no significant differences between the two groups in other measures of change in aortic dimensions, including absolute aortic dimension (Webfigure 3). There was some evidence of heterogeneity in the Z score difference between ARB and β-blockers depending on family history of aortic dissection (favouring ARB in the 110 patients with such a family history, heterogeneity p=0.01), but otherwise no evidence of heterogeneity in any of the other pre-specified sub-groups (Figure 3).

Combining the results of the four trials of ARB versus control with the three trials of ARB versus β-blocker allows for an indirect assessment of β-blocker versus control. With such an analysis, the difference in the annual change in the aortic root Z score between β-blocker and control was -0.09 (95% CI -0.18 to 0.00, p=0.04) (ie, similar to the direct estimate of -0.07 [95% CI -0.12 to -0.01] when comparing ARB with control). Since the trials of an ARB vs control and an ARB vs BB included patients within different age ranges, in a post-hoc exploratory analysis we also examined the indirect comparison of a β-blocker vs control separately within those aged <16 and ≥16 years. We found no evidence that the effect of a β-blocker differed in those aged <16 years compared with those aged ≥16 years (p for heterogeneity 0.09).

We also pre-specified a range of other secondary analyses in our published protocol.^25^ These included assessments of aortic dimension at locations other than the sinuses of Valsalva, assessments using different imaging methods, as well as analyses of haemodynamic variables (e.g. blood pressure) and other physical measurements. The numbers of patients available for such analyses varied, depending on what was included in each trial’s case report forms, and the results are summarised in Webtables 3 and 4. None of these results are qualitatively inconsistent with the main findings. In addition, we pre-specified exploratory analyses using a random effects model: the absolute differences for each of these were similar to those derived from our pre-specified method of analysis (a so called ‘fixed-effect’ analysis), but with wider confidence intervals (as would be expected; Webtable 5). Finally, we pre-specified that for baseline groups defined by age, systolic blood pressure, diastolic blood pressure and body surface area (BSA)-adjusted Z score we would perform additional interaction tests in which these factors were considered as continuous rather than categorical variables. For all 8 interaction tests performed (4 in each of the two groups of trials) there was no good evidence that the effect on aortic root dimension Z score varied significantly depending on the baseline characteristic (the smallest of these interaction p-values was 0.05, which is not statistically significant given the multiple tests performed).

## Discussion

Marfan syndrome affects about 1 in 5000 people, with a global distribution, and affects about 1.6 million people worldwide.^33^ It causes a dramatically increased risk of aortic dissection, at least 100 times greater than the general population,^7^ commonly resulting in premature death or disability. Prophylactic surgery to replace the aortic root is recommended where a large or rapidly expanding aneurysm presents an imminent risk of aortic dissection but such surgery is itself associated with morbidity, occasional mortality, and is not available in all healthcare systems. Effective medical therapy that is well-tolerated by both children and adults could, however, delay or prevent the need for surgery.

This meta-analysis shows that among patients with Marfan syndrome and no prior aortic surgery, an ARB reduces the rate of increase of the aortic root Z score by around one half and that this effect seems to be in addition to any effects of β-blockers (which are discussed below). The robustness of our findings on the effects of an ARB is reinforced by several observations which, since they depended on the availability of individual participant data, went beyond the results of previous meta-analyses of tabular data. ^26–28^ The first of these is that, although in general there was little evidence of heterogeneity of the effect of ARBs among the pre-specified subgroups, ARBs had a significantly greater effect on aortic root dimension Z score among patients with a known pathogenic variant in the *FBN1* gene, which - since *FBN1* variant status is a marker of the certainty of a diagnosis of Marfan syndrome - is what might be expected if an ARB is effective at slowing root expansion in this condition. The second was that all of the pre-specified methods for estimating change in root size yielded statistically significant results, so that our findings were not dependent on the performance of a particular method; this, again, is as might be expected if an ARB is effective.

Our analyses are most informative about the effects of an ARB, since these involved meta-analyses of trials making *direct* comparisons of an ARB versus control. However, they are less definitive for β-blockers, which depended on *indirect* comparisons of two groups of trials (4 comparing an ARB vs control and 3 comparing an ARB versus a β-blocker). Our indirect estimate of the effect of β-blockers is consistent with the results of one small trial that directly compared a β-blocker with no treatment.^10^ However, our estimates rely on the assumption that the effects of ARBs and β-blockers are independent of each other (ie, that the effects of each drug on change in aortic Z score are ‘additive’). Our observation of a lack of significant heterogeneity of the effect of ARBs depending on concomitant use of β-blockers suggests this is a reasonable assumption, but does not prove the assumption to be correct. Since the trials of an ARB vs control and of an ARB vs BB differed substantially in average age at entry (13.9 years for an ARB vs BB and 28.5 for an ARB vs control), we assessed the separate effects of a β-blocker in those aged ≥16 and those aged >16 in stratified analyses. Although we found no evidence that effects of β-blockers varied significantly depending on age, the power to detect any true heterogeneity was limited.

The clinical significance of our results is informed by sample size calculations performed by the Pediatric Heart Network investigators^24^ in which it was assumed that, in an adult Marfan population with mean age 20 and Z-score of 4.3, the threshold for aortic surgery would be reached in about 15 years (when the Z-score = 7.3 and the aortic root diameter = 5.04 cm). Reducing this annual rate of change from +0.20 to +0.12 (i.e. annual reduction of 0.08) would therefore increase the expected time to surgery by about 10 years (since, at an annual increase in Z-score of +0.12 rather than +0.20, it would take 25 rather than 15 years for the Z-score to increase from 4.3 to 7.3). Such an annual reduction is consistent with the absolute changes in Z-score in our analyses (-0.07 for an ARB and -0.09 for a beta-blocker).

A limitation of our analysis is that despite making every effort to obtain all available trial datasets, not all were available for individual data analysis: one published trial^16^ did not contribute data but was very small (34 participants followed for only 6 months) and as its conclusions were consistent with the results of the meta-analysis it would not have influenced our conclusions. More significantly, a trial of moderate size (n=262, follow up 48 months), was not available from the investigators.^32^ The main findings of that trial have, however, been reported in abstract form and showed that the combination of an ARB (losartan) and a β-blocker (nebivolol) reduced progression as compared to either treatment alone (p=0.009),^32^ which is consistent with the main findings of the meta-analysis. Among the trials included in our meta-analysis, one used irbesartan^23^ whilst losartan was used in the others, hence the amount of data available for irbesartan was very limited compared with losartan. None randomly allocated participants to pre-specified ARB dosing strategies or to different agents. Consequently, it was not possible to explore whether any particular ARB selection or dosing strategy was superior to any other (and similar limitations apply to β-blockers). The generalisability of our findings to older Marfan patients is also uncertain, as only 11% of the randomised patients were aged over ≥40 years while only 6% were aged ≥50 years. Finally, even in this meta-analysis of all eligible and available trials, the number of patients experiencing major clinical outcomes was too small to provide sufficient statistical power to detect benefit on such outcomes over the relatively short duration of the trials.

In summary, in these trials of patients with Marfan syndrome, ARBs reduced the rate of enlargement of the aortic root by about one half, including among those already taking a β-blocker. The effect was particularly large among patients with a pathogenic *FBN1* variant, strengthening the main finding. The effects of β-blockers were similar in magnitude to those of ARBs. Moreover, for ARB versus control, there was no evidence that the effect size depended on use of β-blockers. Our findings therefore suggest that, if tolerated, the combination of a β-blocker and ARB would reduce the rate of enlargement of the aortic root by at least one half, and potentially by much more than this which, if maintained over a sustained period, would be expected to delay the need for surgery substantially.

## Supplementary Material

Appendix

## Figures and Tables

**Table 1:** Characteristics of ten trials of angiotensin receptor blockers in patients with Marfan syndrome

Trial	Treatment comparison (daily doses)	Number of patients randomised	Median age, years	Median follow-up, months	Measures collected, months	Data provided to MTT secretariat	Main outcome measures					
							Body Surface Area		Aortic dimension at the SV (mm)		Z score at the SV	
							Method	Baseline mean (SD)	Method(s)	Baseline mean (SD)	Method	Baseline mean (SD)
**ARB vs control (placebo or open control)**															
Marfan-Sartan^18^	Losartan (50-100 mg) vs Placebo	299	26	42	0, 6, 12, 18, 24, 30, 36, 42, 48, 54, 60	Yes	^†^	1.83 (0.27)	Echo/End diastole/Leading edge to leading edge	38.8 (5.8)	Roman	3.60 (2.15)
COMPARE^20^	Losartan (50-100 mg) vs open control	233	35	36	0, 36	Yes	Haycock	2.02 (0.24)	MRI/End diastole/Inner edge to outer edge	44.3 (5.2)	^‡^	4.42 (1.75)
AIMS^23^	Irbesartan (150-300 mg) vs placebo	192	18	46	0, 12, 24, 36, 48, 60	Yes	DuBois	1.71 (0.40)	Echo/Peak Systole/Inner edge to inner edge	34.4 (5.6)	Devereux	3.24 (2.04)
Ghent Marfan^15^	Losartan (25-100 mg) vs placebo	22	36	36	0, 6, 12, 24, 36	Yes	DuBois	1.98 (0.24)	Echo/Leading edge to leading edge	41.2 (3.5)	^‡^	3.55 (1.04)
Taiwan^22^	Losartan plus β-blocker vs β-blocker^¶^	28	13	35	0, 6, 12, 18, 24, 30, 35	Yes (but only included in sensitivity analyses)	Unknown	1.39 (0.38)	Echo/Inner edge to inner edge	32.9 (6.1)	^‡^	2.07 (1.89)
_Italy_^19^	Losartan (100 mg adults or ≤1.4 mg/kg children) plus nebivolol (10 mg adults or ≤0.16 mg/kg children) vs nebivolol (10 mg adults or ≤0.16 mg/kg children)	160^§^	Unknown	48	0, 12, 24, 36	No (trial yet to publish its full results)	Unknown	-	-	-	-	-
**ARB vs β-blocker**															
PHN^24^	Losartan (0.4-1.4 mg/kg) vs atenolol (0.5-4 mg/kg)	608	11	36	0, 6, 12, 24, 36	Yes	Haycock	1.28 (0.48)	Echo/Systole/Inner edge to inner edge	33.6 (7.1)	Sluysmans	4.32 (1.35)
LOAT^21^	Losartan (12.5-100 mg) vs atenolol (12.5-100 mg)	140	26	36	0, 36	Yes	DuBois	1.75 (0.36)	MRI/End diastolic frame	36.1 (6.2)	Devereux	3.17 (2.21)
Canada^17^	Losartan (25 mg) vs atenolol (25-50 mg)	18	17	12	0, 3, 6, 9, 12	Yes	DuBois	1.77 (0.22)	Echo/Trailing edge to leading edge	39.0 (5.8)	^‡^	3.55 (1.93)
_Italy_^19^	Losartan (100 mg adults or ≤1.4 mg/kg children) vs nebivolol (10 mg adults or ≤0.16 mg/kg children)	155^§^	Unknown	48	0, 12, 24, 36, 48	No (trial yet to publish its full results)	Unknown	-	-	-	-	-
Boston^16^	Losartan (100 mg) vs atenolol (50 mg)	34	35	6	0, 6	No (trialist unable to provide data)	Unknown	-	-	-	-	-

For each trial, the imaging method used to estimate aortic root dimension was the method used in that trial’s primary analysis.

† Data not provided by the trialist so estimated using the DuBois^34^ method (ie, BSA[m^2^] = weight (kg)^0.425^ X height (cm)^0.725^ X 0.007184).

‡ Data not provided by the trialist so estimated using the Devereux^35^ method (ie, Value[cm] –(2.423+0.009age + 0.461BSA –0.267sex[1=M, 2=F])/0.261).

¶ Adults were randomised to losartan (25-100 mg/day)plus β-blocker (at a ‘low maintenance dose’ [50 mg atenolol once daily or 20 mg propranolol twice daily]) versus β-blocker (atenolol or propranolol, maximum 150 mg/day), while children were randomised to losartan (0.7 mg/kg/day, to a maximum of 50 mg/day) plus β-blocker (‘low maintenance dose’, 1 mg/kg/day atenolol or propranolol) vs β-blocker (atenolol or propranolol, maximum 2 mg/kg/day).

§ Number reported in trialists’ abstract was number analysed (the total number of patients randomised across all three arms of the Italian trial was 262).

**Table 2:** Baseline characteristics by randomised allocation

	ARB vs Control		ARB vs β-blocker	
	ARB (*n*=353)	Control (*n*=323)	ARB (*n*=384)	β-blocker (*n*=382)
Median follow-up, years	3.0	3.0	3.0	3.0
Age, years	28.8 (14.7)	28.3 (13.8)	13.9 (9.9)	13.9 (9.7)
<16	75 (21%)	67 (21%)	258 (67%)	254 (66%)
≥16 to <25	80 (23%)	78 (24%)	82 (21%)	88 (23%)
≥25 to <40	114 (32%)	119 (37%)	37 (10%)	31 (8%)
≥40	84 (24%)	59 (18%)	7 (2%)	9 (2%)
Gender				
Male	164 (46%)	145 (45%)	218 (57%)	212 (55%)
Female	189 (54%)	178 (55%)	166 (43%)	170 (45%)
Family history of Marfan syndrome				
Yes	100 (28%)	82 (25%)	187 (49%)	188 (49%)
No	164 (46%)	158 (49%)	111 (29%)	116 (30%)
Unknown	89 (25%)	83 (26%)	86 (22%)	78 (20%)
Family history of aortic dissection				
Yes	6 (2%)	1 (<0.5%)	55 (14%)	58 (15%)
No	4 (1%)	4 (1%)	258 (67%)	254 (66%)
Unknown	343 (97%)	318 (98%)	71 (18%)	70 (18%)
Presence of *FBN1*				
Yes	270 (76%)	256 (79%)	135 (35%)	145 (38%)
No	59 (17%)	45 (14%)	27 (7%)	20 (5%)
Unknown	24 (7%)	22 (7%)	222 (58%)	217 (57%)
Ectopia lentis				
Yes	84 (24%)	67 (21%)	129 (34%)	133 (35%)
No	116 (33%)	108 (33%)	150 (39%)	134 (35%)
Unknown	153 (43%)	148 (46%)	105 (27%)	115 (30%)
Current β-blocker use	265 (75%)	242 (75%)	0 (0%)	0 (0%)
Aorta at the Sinuses of Valsalva				
Dimension, mm	39.0 (6.8)	38.9 (6.5)	34.2 (7.0)	34.2 (7.1)
Z score	3.76 (2.14)	3.64 (1.94)	4.18 (1.71)	4.03 (1.50)
Other baseline measures				
Weight, kg	67.6 (19.7)	69.6 (21.3)	45.0 (23.7)	46.5 (23.7)
Height, cm	178 (15)	179 (15)	155 (31)	156 (32)
Systolic blood pressure, mmHg	117 (16)	117 (15)	102 (16)	102 (16)
Diastolic blood pressure, mmHg	70 (11)	70 (10)	62 (11)	62 (11)
Heart rate, beats/min	64 (14)	65 (14)	78 (18)	77 (17)
Body surface area, m^2^	1.83 (0.32)	1.86 (0.33)	1.36 (0.49)	1.40 (0.50)
Body mass index, kg/m^2^	20.9 (4.6)	21.3 (5.3)	17.3 (4.0)	17.5 (4.0)

Results are count (%), median or mean (standard deviation). 70 patients with prior aortic root surgery at enrolment are excluded: 2 (ARB) vs 5 (placebo) from Ghent Marfan and 27 (ARB) vs 36 (control) patients from COMPARE.

## Data Availability

The NIH/NHLBI Pediatric Heart Network Marfan Study dataset was used in preparation of this work. Data were downloaded from http://pediatricheartnetwork.org/ForResearchers/PHNPublicUseDatasets.aspx on 15/10/2019. Other individual level datasets were provided to the MTT secretariat in Oxford by the trialists. Data sharing requests for these trials would need to be sent to the principal investigators of the trials.

## Research in Context

### Evidence before this study

Searches of the literature identified a number of randomised trials of an ARB vs placebo (or open control) in which the aim was to estimate the effects of treatment with ARBs on aortic root size in patients with Marfan syndrome. Several meta-analyses of published data from these trials were also identified, but none was able to define the effects of ARBs in different circumstances, including according to age, sex or blood pressure, but also according to whether a β-blocker was part of existing treatment, and whether a diagnosis of Marfan syndrome had been confirmed by genotyping. There were no large, randomised trials assessing the efficacy of a β-blocker for Marfan syndrome, despite the fact that such treatment is used widely in this condition.

### Added Value of this study

This meta-analysis of individual patient data from randomised trials, which followed a protocol that was agreed and published prior to any analyses being conducted, showed that ARBs reduced the rate of aortic root enlargement by about one half, and that this effect was generalizable to different types of patients. In particular, ARBs were effective even among those already taking a β-blocker. The estimated effect of ARBs was significantly greater among those with a pathogenic *FBN1* variant, providing biological support for the effect being real. β-blockers were estimated to have a similar beneficial effect as ARBs.

### Implications of all the available evidence

If tolerated, the combination of a β-blocker and an ARB may reduce the rate of enlargement of the aortic root by at least one half, and potentially by much more than this. Although this meta-analysis of trials did not have sufficient power to assess effects on the need for surgery (and it is unlikely that any randomised study will ever be conducted to study this question directly), as elective surgery is almost always driven by aortic root size and rate of expansion, our results suggest that long term combination therapy may eventually reduce such outcomes.
