# Angiotensin receptor blockers and β blockers in Marfan syndrome: an individual patient data meta-analysis of randomised trials

**DOI:** 10.1016/S0140-6736(22)01534-3

**Published:** 2022-09-10

**Authors:** Alex Pitcher, Enti Spata, Jonathan Emberson, Kelly Davies, Heather Halls, Lisa Holland, Kate Wilson, Christina Reith, Anne H Child, Tim Clayton, Matthew Dodd, Marcus Flather, Xu Yu Jin, George Sandor, Maarten Groenink, Barbara Mulder, Julie De Backer, Arturo Evangelista, Alberto Forteza, Gisela Teixido-Turà, Catherine Boileau, Guillaume Jondeau, Olivier Milleron, Ronald V Lacro, Lynn A Sleeper, Hsin-Hui Chiu, Mei-Hwan Wu, Stefan Neubauer, Hugh Watkins, Hal Dietz, Colin Baigent, The Marfan Treatment Trialists’ Collaboration

**Affiliations:** a The Heart Centre, John Radcliffe Hospital, Oxford University Hospitals NHS Foundation Trust, Oxford, UK; b MRC Population Health Research Unit, Nuffield Department of Population Health, University of Oxford, Oxford, UK; c Clinical Trial Service Unit and Epidemiological Studies Unit, Nuffield Department of Population Health, University of Oxford, Oxford, UK; d Royal Brompton and Harefield Hospitals Unit, Guy's and St Thomas’ NHS Trust and Department of Surgery and Oncology, Imperial College London, London, UK; e Clinical Trials Unit, Department of Medical Statistics, London School of Hygiene & Tropical Medicine, London, UK; f Faculty of Medicine and Health Sciences, University of East Anglia, Norwich, UK; g Children's Heart Centre, British Columbia's Children's Hospital, Vancouver, BC, Canada; h Department of Paediatrics, Faculty of Medicine, University of British Columbia, Vancouver, BC, Canada; i Academic Medical Center, University of Amsterdam, Amsterdam, Netherlands; j Center for Medical Genetics and Department of Cardiology, Ghent University Hospital, Ghent, Belgium; k Servei de Cardiologia, Hospital Universitari Vall d'Hebron, Barcelona, Spain; l Hospital Puerta de Hierro, Majadahonda, Spain; m Department of Cardiology, Hospital Universitari Vall d’Hebron, CIBER-CV, Vall d’Hebron institut de Recerca, Universitat Autònoma de Barcelona, Barcelona, Spain; n Université Paris Cité and Université Sorbonne Paris Nord, Inserm U1148, LVTS, F-75018 Paris, France; o Service de Cardiologie, AP-HP Hôpital Bichat-Claude Bernard, F-75018, Paris, France; p CRMR Syndrome de Marfan et apparentés. AP-HP Hôpital Bichat-Claude Bernard, F-75018, Paris, France; q Department of Cardiology, Boston Children's Hospital, Boston, MA, USA; r Department of Pediatrics, Harvard Medical School, Boston, MA, USA; s Department of Pediatrics, Taipei Tzu Chi Hospital, Buddhist Tzu Chi Medical Foundation, New Taipei City, Taiwan; t Department of Pediatrics and Adult Congenital Heart Center, National Taiwan University Hospital, Taipei, Taiwan; u Oxford Centre for Clinical Magnetic Resonance Research, Division of Cardiovascular Medicine, University of Oxford, Oxford, UK; v Radcliffe Department of Medicine, University of Oxford, Oxford, UK; w Howard Hughes Medical Institute and Department of Genetic Medicine, Johns Hopkins University School of Medicine, Baltimore, MD, USA; x European Reference Network for Rare Multisystemic Vascular Disease (VASCERN), HTAD Rare Disease Working Group, Amsterdam, The Netherlands; y European Reference Network for Rare Multisystemic Vascular Disease (VASCERN), HTAD Rare Disease Working Group, Ghent, Belgium; z European Reference Network for Rare Multisystemic Vascular Disease (VASCERN), HTAD Rare Disease Working Group, Barcelona, Spain; aa European Reference Network for Rare Multisystemic Vascular Disease (VASCERN), HTAD Rare Disease Working Group, Paris, France

## Abstract

**Background:**

Angiotensin receptor blockers (ARBs) and β blockers are widely used in the treatment of Marfan syndrome to try to reduce the rate of progressive aortic root enlargement characteristic of this condition, but their separate and joint effects are uncertain. We aimed to determine these effects in a collaborative individual patient data meta-analysis of randomised trials of these treatments.

**Methods:**

In this meta-analysis, we identified relevant trials of patients with Marfan syndrome by systematically searching MEDLINE, Embase, and CENTRAL from database inception to Nov 2, 2021. Trials were eligible if they involved a randomised comparison of an ARB versus control or an ARB versus β blocker. We used individual patient data from patients with no prior aortic surgery to estimate the effects of: ARB versus control (placebo or open control); ARB versus β blocker; and indirectly, β blocker versus control. The primary endpoint was the annual rate of change of body surface area-adjusted aortic root dimension Z score, measured at the sinuses of Valsalva.

**Findings:**

We identified ten potentially eligible trials including 1836 patients from our search, from which seven trials and 1442 patients were eligible for inclusion in our main analyses. Four trials involving 676 eligible participants compared ARB with control. During a median follow-up of 3 years, allocation to ARB approximately halved the annual rate of change in the aortic root Z score (mean annual increase 0·07 [SE 0·02] ARB *vs* 0·13 [SE 0·02] control; absolute difference –0·07 [95% CI –0·12 to –0·01]; p=0·012). Prespecified secondary subgroup analyses showed that the effects of ARB were particularly large in those with pathogenic variants in fibrillin-1, compared with those without such variants (heterogeneity p=0·0050), and there was no evidence to suggest that the effect of ARB varied with β-blocker use (heterogeneity p=0·54). Three trials involving 766 eligible participants compared ARBs with β blockers. During a median follow-up of 3 years, the annual change in the aortic root Z score was similar in the two groups (annual increase –0·08 [SE 0·03] in ARB groups *vs* –0·11 [SE 0·02] in β-blocker groups; absolute difference 0·03 [95% CI –0·05 to 0·10]; p=0·48). Thus, indirectly, the difference in the annual change in the aortic root Z score between β blockers and control was –0·09 (95% CI –0·18 to 0·00; p=0·042).

**Interpretation:**

In people with Marfan syndrome and no previous aortic surgery, ARBs reduced the rate of increase of the aortic root Z score by about one half, including among those taking a β blocker. The effects of β blockers were similar to those of ARBs. Assuming additivity, combination therapy with both ARBs and β blockers from the time of diagnosis would provide even greater reductions in the rate of aortic enlargement than either treatment alone, which, if maintained over a number of years, would be expected to lead to a delay in the need for aortic surgery.

**Funding:**

Marfan Foundation, the Oxford British Heart Foundation Centre for Research Excellence, and the UK Medical Research Council.

## Introduction

Marfan syndrome is a genetic disorder, usually caused by pathogenic variants in the fibrillin-1 *(FBN1)* gene that causes progressive enlargement of the aortic root. If unchecked, aortic enlargement in Marfan syndrome can lead to life-threatening aortic dissection, sometimes in early adulthood.^1^, ^2^, ^3^, ^4^, ^5^, ^6^, ^7^ Initial treatment is aimed at slowing aortic root growth, and β blockers are widely used for this purpose, but their use is based mainly on the results of observational studies^8^, ^9^ and one small randomised trial.^10^

The discovery that transforming growth factor β (TGFβ) dysregulation is implicated in the pathogenesis of some aortic aneurysms led to the hypothesis that angiotensin receptor blockade (which attenuates TGFβ activity) might slow aortic root growth in Marfan syndrome.^11^, ^12^ Favourable results in animal models^11^ and in small observational studies in humans^12^, ^13^, ^14^ led to several randomised trials in patients with Marfan syndrome, the first of which was published in 2013.^15^, ^16^, ^17^, ^18^, ^19^, ^20^, ^21^, ^22^, ^23^, ^24^ Combining these trials could increase the precision in estimates of treatment effect and increase the power of subgroup analyses. In 2012, we established a collaborative group (the Marfan Treatment Trialists’ [MTT] Collaboration) to do a meta-analysis of individual patient data from all relevant Marfan syndrome trials. A protocol was subsequently agreed for the rationale, design, and conduct of the meta-analysis.^25^

**Research in context**

**Evidence before this study**

We searched Embase, MEDLINE, and CENTRAL from inception until Nov 2, 2021, for randomised trials that assessed the effects of angiotensin receptor blocker (ARB) versus control or ARB versus β blockers in patients with Marfan syndrome. Cochrane search filters were used in Embase and Medline to identify randomised trials, and the terms “Marfan” or “Marfan syndrome” were used in all three databases (appendix p 2). The searches were not restricted to English language publications. We identified a number of randomised trials of ARB versus placebo (or open control) in which the aim was to estimate the effects of treatment with ARBs on aortic root size in patients with Marfan syndrome. Several meta-analyses of published data from these trials were also identified, but none were able to define the effects of ARBs in different circumstances, including according to age, sex, or blood pressure, but also according to whether or not a β blocker was part of existing treatment, and whether or not a diagnosis of Marfan syndrome had been confirmed by genotyping. There were no large, randomised trials assessing the efficacy of a β blocker for Marfan syndrome, despite the fact that such treatment is used widely for this condition.

**Added value of this study**

This meta-analysis of individual patient data from randomised trials, which followed a protocol that was agreed and published before any analyses, showed that ARBs reduced the rate of aortic root enlargement by about one half, and that this effect was generalisable to different types of patients. In particular, ARBs were effective even among those already taking a β blocker. The estimated effect of ARBs was significantly greater among those with a pathogenic variant in fibrillin-1, than those without such a fibrillin-1 variant, providing biological support for the effect. β blockers were estimated to have a similar beneficial effect as ARBs.

**Implications of all the available evidence**

If tolerated, the combination of a β blocker and an ARB could reduce the rate of enlargement of the aortic root by at least one half, and potentially by much more than this. Although this meta-analysis of trials did not have sufficient power to assess effects on the need for surgery (and it is unlikely that any randomised study will ever be done to study this question directly), as elective surgery is almost always driven by aortic root size and rate of expansion, our results suggest that long-term combination therapy could eventually reduce such outcomes.

Since 2012, most of the participating trial groups in the MTT Collaboration have reported their results,^15^, ^16^, ^17^, ^18^, ^20^, ^21^, ^22^, ^23^, ^24^ and several meta-analyses of published data have been reported.^26^, ^27^, ^28^ Two of these meta-analyses^26^, ^28^ did not include the recent UK-based AIMS trial,^23^ but one that did include this trial^27^ concluded that angiotensin receptor blockers (ARBs) are effective when used alone or when added to a β blocker. Since this meta-analysis included only published data, it was subject to biases that might arise from selective reporting of findings in publications, and it was not able to harmonise definitions of aortic root size for all trials or explore treatment effects in detail (eg, among particular prognostic subgroups such as those with a confirmed *FBN1* pathogenic variant). The present report describes a meta-analysis in which the availability of individual patient data removes these limitations, allowing a more complete assessment of ARBs in Marfan syndrome. By prespecifying the use of indirect comparisons of trials of an ARB versus control and of an ARB versus β blocker, our report also provides an assessment of the effects of β-blocker therapy given alone and the effects of combined ARB and β-blocker therapy.

## Methods

### Study design and outcomes

In this meta-analysis, we identified relevant trials by systematically searching MEDLINE, Embase, and CENTRAL from database inception to Nov 2, 2021. Trials were eligible if they involved a randomised comparison of an ARB versus control or an ARB versus β blocker in patients with Marfan syndrome and if patient-level data were available. A description of the search terms is in the appendix (p 2). The review of search results was done independently by two authors (including HH, KD, KW, and LH) and adjudicated by either AP or CBa. Relevant trials were also sought through enquiry with authors of collaborating trials.^25^ Each trial was reviewed for eligibility by reviewing its protocol and methods, and clarification was sought as necessary by discussion with the authors of the relevant trial. Bias was accounted for by including only properly randomised, unconfounded trials in the main analyses. Sources of variability within and between studies was controlled by a harmonised definition of patients to be included in the analyses, and explored by prespecified subgroup analyses. The protocol for our study has been published.^25^ The primary aims of this meta-analysis were to estimate the effects of ARB and β blockers on the change in aortic root size in patients with Marfan syndrome and no previous aortic root surgery. Thus, patients with previous aortic root surgery in the identified trials were excluded from analyses. The primary comparisons involved only the unconfounded trials (ie, trials that had no protocol-mandated differences between randomised groups other than those created by the randomised allocations), but a sensitivity analysis included one trial^22^ in which there were different dosing strategies for β blockers in the ARB and control groups of the study. The prespecified primary outcome was the annual rate of change of body surface area (BSA)-adjusted aortic root dimension Z score, measured at the sinuses of Valsalva. The secondary outcome was the annual rate of change of the absolute aortic root dimension measured at the sinuses of Valsalva. Other secondary aims were to assess those effects across different subgroups defined on the basis of patients’ characteristics at baseline; to assess effects on cardiovascular outcomes, including aortic dissection, aortic root surgery and death, as well as the composite of these three outcomes; and to assess effects on a range of other outcomes and measures that were sufficiently complete to permit meaningful analyses (appendix p 4).

### Data analysis

We requested individual participant data on relevant characteristics including age, sex, previous aortic surgery, family history of Marfan syndrome, pathogenic variants in relevant genes, ectopia lentis, β-blocker usage, physical measurements, and blood pressure. We also collected data on aortic measurements, surgery, dissection, and death during the trial. The data analysis methods used in our study have been published elsewhere.^25^ Z scores were calculated using the method used by each trial (as reported in their main analysis) except in cases in which values were provided directly by the trialist. Secondary analyses using the methods of Campens and colleagues^29^ and Pettersen and colleagues^30^ were done to explore the effect of using an alternative method to estimate aortic root Z score. A two-stage meta-analysis approach was used. For each patient, a linear slope of the annual rate of change (from baseline) of the BSA-adjusted Z score was calculated. The difference in mean slopes between treatment groups (and its SE) was then calculated for each trial and standard inverse-variance-weighted methods were used to estimate the overall inverse-variance-weighted average difference in slopes across all trials. A random effects meta-analysis, which assumes that the underlying set of trials are representative of an underlying population of possible trial designs, was also done as a sensitivity analysis. Patients with missing data on rate of change in aortic root Z score were excluded. To allow for multiple subdivisions of the data, only summary effect estimates are presented with 95% CIs; all other effect estimates (such as results from individual trials or in particular patient subgroups) are presented with 99% CIs.

An indirect assessment of the effect of β blockers compared with controls was calculated using indirect comparisons of trial results^31^ as follows: if d_1_ (with variance v_1_) is the difference in mean annual rate of change in aortic root Z scores estimated from the three trials that compared ARB with β blockers and d_2_ (with variance v_2_) is the difference in mean annual rate of change in aortic root Z scores estimated from the four trials that compared ARB with controls, then an indirect estimate of the effect of β blockers is provided by d_2_ – d_1_ (which has variance equal to v_1_ + v_2_). This indirect analysis assumes that the effects of ARBs and β blockers are additive (that is, the effect of an ARB is the same if a β blocker is given or not and the effect of a β blocker is the same irrespective of whether an ARB is given or not).

For the primary comparisons, a two-sided p value less than 0·05 was considered significant. Analyses were done using SAS version 9.4 and R version 3.5.0. The MTT database is a research database, and did not involve accessing or otherwise processing patient-identifiable information, and hence did not require ethical approval.

### Role of the funding source

The funders of the study had no role in study design, data collection, data analysis, data interpretation, writing of the report, or the decision to submit.

## Results

We identified ten trials with a total of 1836 patients as being potentially eligible for the study (table 1; appendix p 7).^15^, ^16^, ^17^, ^18^, ^19^, ^20^, ^21^, ^22^, ^23^, ^24^ Three trials, with a total of 324 patients, were not included in the primary analyses: one trial (262 patients) was published as an abstract only and was unable to contribute data;^32^ one trial (34 patients) was published but unable to contribute data;^16^ and one trial (28 patients) contributed data but was found to be confounded owing to protocol-mandated adjustment in β-blocker doses in the control group^22^ (this trial contributed only to a sensitivity analysis). Of the seven remaining trials, 70 (4·6%) of the 1512 randomised patients were excluded because they had had previous aortic root surgery. The main analyses therefore include individual data from 1442 participants in seven trials.^15^, ^17^, ^18^, ^20^, ^21^, ^22^, ^23^, ^24^

**Table 1:** Characteristics of ten trials of angiotensin receptor blockers in patients with Marfan syndrome

	**Treatment comparison (daily doses)**	**Number of randomly assigned patients**	**Median age, years**	**Median follow-up, months**	**Measures collected, months**	**Data provided to MTT secretariat**	**Main outcome measures**					
							Body surface area		Aortic dimension at the sinuses of Valsalva (mm)		Z score at the sinuses of Valsalva	
							Method	Baseline, mean (SD)	Methods	Baseline, mean (SD)	Method	Baseline, mean (SD)
**ARB *vs* control (placebo or open control)**												
Marfan Sartan^18^	Losartan (50–100 mg) *vs* placebo	299	26	42	0, 6, 12, 18, 24, 30, 36, 42, 48, 54, 60	Yes	*	1·83 (0·27)	Echo, end diastole, and leading edge to leading edge	38·8 (5·8)	Roman and colleagues^33^	3·60 (2·15)
COMPARE^20^	Losartan (50–100 mg) *vs* open control	233	35	36	0, 36	Yes	Haycock and colleagues^34^	2·02 (0·24)	MRI, end diastole, and inner edge to outer edge	44·3 (5·2)	†	4·42 (1·75)
AIMS^23^	Irbesartan (150–300 mg) *vs* placebo	192	18	46	0, 12, 24, 36, 48, 60	Yes	DuBois and Du Bois^35^	1·71 (0·40)	Echo, peak systole, and inner edge to inner edge	34·4 (5·6)	Devereux and colleagues^36^	3·24 (2·04)
Ghent Marfan^15^	Losartan (25–100 mg) *vs* placebo	22	36	36	0, 6, 12, 24, 36	Yes	DuBois and Du Bois^35^	1·98 (0·24)	Echo and leading edge to leading edge	41·2 (3·5)	†	3·55 (1·04)
Taiwanese trial^22^	Losartan plus β blocker *vs* β blocker‡	28	13	35	0, 6, 12, 18, 24, 30, 35	Yes (but only included in sensitivity analyses)	Unknown	1·39 (0·38)	Echo and inner edge to inner edge	32·9 (6·1)	†	2·07 (1·89)
Italian trial^19^	Losartan (100 mg adults or ≤1·4 mg/kg children) plus nebivolol (10 mg adults or ≤0·16 mg/kg children) *vs* nebivolol (10 mg adults or ≤0·16 mg/kg children)	160§	Unknown	48	0, 12, 24, 36, 48	No (trial yet to publish its full results)	Unknown	..	..	..	..	..
**ARB *vs* β blocker**												
PHN^24^	Losartan (0·4–1·4 mg/kg) *vs* atenolol (0·5–4·0 mg/kg)	608	11	36	0, 6, 12, 24, 36	Yes	Haycock and colleagues^34^	1·28 (0·48)	Echo, systole, and inner edge to inner edge	33·6 (7·1)	Sluysmans and Colan^37^	4·32 (1·35)
LOAT^21^	Losartan (12·5–100 mg) *vs* atenolol (12·5–100 mg)	140	26	36	0, 36	Yes	DuBois and Du Bois^35^	1·75 (0·36)	MRI and end diastolic frame	36·1 (6·2)	Devereux and colleagues^36^	3·17 (2·21)
Canadian trial^17^	Losartan (25 mg) *vs* atenolol (25–50 mg)	18	17	12	0, 3, 6, 9, 12	Yes	DuBois and Du Bois^35^	1·77 (0·22)	Echo and trailing edge to leading edge	39·0 (5·8)	†	3·55 (1·93)
Italian trial^19^	Losartan (100 mg adults or ≤1·4 mg/kg children) *vs* nebivolol (10 mg adults or ≤0·16 mg/kg children)	155§	Unknown	48	0, 12, 24, 36, 48	No (trial yet to publish its full results)	Unknown	..	..	..	..	..
Boston, USA trial^16^	Losartan (100 mg) *vs* atenolol (50 mg)	34	35	6	0, 6	No (trialist unable to provide data)	Unknown	..	..	..	..	..

For each trial, the imaging method used to estimate aortic root dimension was the method used in that trial's primary analysis. ARB=angiotensin receptor blocker. MTT=Marfan Treatment Trialists.

* Data not provided by the trialist so estimated using the DuBois and Du Bois^34^ method.

† Data not provided by the trialist so estimated using the Devereux and colleagues^35^ method).

‡ Adults were randomly assigned to losartan (25–100 mg per day) plus β blocker (at a low maintenance dose [50 mg atenolol once daily or 20 mg propranolol twice daily]) *vs* β blocker (atenolol or propranolol, maximum 150 mg per day), and children were randomly assigned to losartan (0·7 mg/kg per day, to a maximum of 50 mg per day) plus β blocker (low maintenance dose, 1 mg/kg per day atenolol or propranolol) *vs* β blocker (atenolol or propranolol, maximum 2 mg/kg per day).

§ Number reported in trialists' abstract was number analysed (the total number of patients randomly assigned across all three groups of the Italian trial was 262).

**Figure 1:**
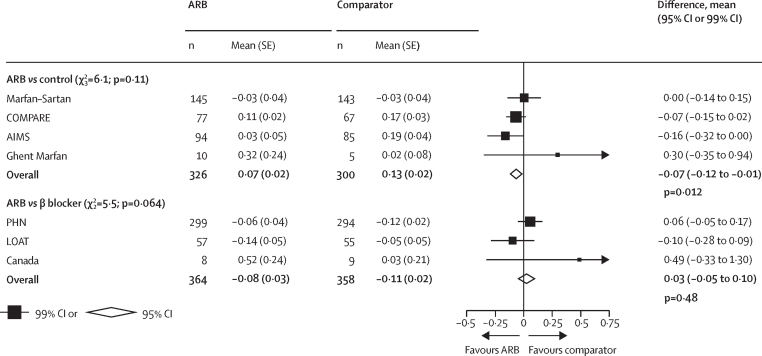
Annual rate of change of body surface area-adjusted aortic root dimension Z score at the sinuses of Valsalva Indirect effect of β blocker *vs* control is –0·09 (95% CI –0·18 to 0·00); p value=0·042 (β blocker minus control). ARB=angiotensin receptor blocker.

Data for the primary analysis were available from four trials of ARB versus control, including 676 patients (excluding 70 patients with previous aortic root surgery at enrolment; 353 assigned to ARB and 323 to control).^15^, ^18^, ^20^, ^23^ The mean age of participants in these trials was 29 years (SD 14), 367 (54%) were female and 507 (75%) were receiving β blockers at baseline (all trials allowed patients to remain on their β blockers). Overall, 526 (83%) of 630 genotyped individuals had an *FBN1* pathogenic variant (table 2). The mean Z score at the sinuses of Valsalva at baseline was 3·76 (SD 2·14) in patients allocated to ARB and 3·64 (SD 1·94) in patients allocated to control. The mean annual change of the Z score during follow-up was 0·07 (SE 0·02) in the ARB group and 0·13 (SE 0·02) in the control group, corresponding to a mean difference of –0·07 (95% CI –0·12 to –0·01; p=0·012; figure 1), which represents an approximate halving in the annual rate of change in the aortic root Z score in the ARB group compared with the control group.

**Table 2:** Baseline characteristics by randomised allocation

		**ARB *vs* control**		**ARB *vs* β blocker**	
		ARB (n=353)	Control (n=323)	ARB (n=384)	β blocker (n=382)
Median follow-up, years		3·0 (2·9–4·0)	3·0 (3·0–4·0)	3·0 (3·0–3·0)	3·0 (3·0–3·0)
Mean age, years		28·8 (14·7)	28·3 (13·8)	13·9 (9·9)	13·9 (9·7)
Age, years					
	<16	75 (21%)	67 (21%)	258 (67%)	254 (66%)
	≥16 to <25	80 (23%)	78 (24%)	82 (21%)	88 (23%)
	≥25 to <40	114 (32%)	119 (37%)	37 (10%)	31 (8%)
	≥40	84 (24%)	59 (18%)	7 (2%)	9 (2%)
Gender					
	Male	164 (46%)	145 (45%)	218 (57%)	212 (55%)
	Female	189 (54%)	178 (55%)	166 (43%)	170 (45%)
Family history of Marfan syndrome					
	Yes	100 (28%)	82 (25%)	187 (49%)	188 (49%)
	No	164 (46%)	158 (49%)	111 (29%)	116 (30%)
	Unknown	89 (25%)	83 (26%)	86 (22%)	78 (20%)
Family history of aortic dissection					
	Yes	6 (2%)	1 (<1%)	55 (14%)	58 (15%)
	No	4 (1%)	4 (1%)	258 (67%)	254 (66%)
	Unknown	343 (97%)	318 (98%)	71 (18%)	70 (18%)
Presence of *FBN1*					
	Yes	270 (76%)	256 (79%)	135 (35%)	145 (38%)
	No	59 (17%)	45 (14%)	27 (7%)	20 (5%)
	Unknown	24 (7%)	22 (7%)	222 (58%)	217 (57%)
Ectopia lentis					
	Yes	84 (24%)	67 (21%)	129 (34%)	133 (35%)
	No	116 (33%)	108 (33%)	150 (39%)	134 (35%)
	Unknown	153 (43%)	148 (46%)	105 (27%)	115 (30%)
Current β-blocker use		265 (75%)	242 (75%)	0	0
Aorta at the sinuses of Valsalva					
	Mean dimension, mm	39·0 (6·8)	38·9 (6·5)	34·2 (7·0)	34·2 (7·1)
	Mean Z score	3·76 (2·14)	3·64 (1·94)	4·18 (1·71)	4·03 (1·50)
Other baseline measures					
	Mean weight, kg	67·6 (19·7)	69·6 (21·3)	45·0 (23·7)	46·5 (23·7)
	Mean height, cm	178 (15)	179 (15)	155 (31)	156 (32)
	Mean systolic blood pressure, mm Hg	117 (16)	117 (15)	102 (16)	102 (16)
	Mean diastolic blood pressure, mm Hg	70 (11)	70 (10)	62 (11)	62 (11)
	Mean heart rate, beats per min	64 (14)	65 (14)	78 (18)	77 (17)
	Mean body surface area, m^2^	1·83 (0·32)	1·86 (0·33)	1·36 (0·49)	1·40 (0·50)
	Mean BMI, kg/m^2^	20·9 (4·6)	21·3 (5·3)	17·3 (4·0)	17·5 (4·0)

Data are n (%), median (IQR), or mean (SD). 70 patients with previous aortic root surgery at enrolment were excluded: two in the ARB group and five in the placebo group from Ghent Marfan and 27 in the ARB group and 36 in the control group from COMPARE. ARB=angiotensin receptor blocker. *FBN1*=fibrillin-1.

**Figure 2:**
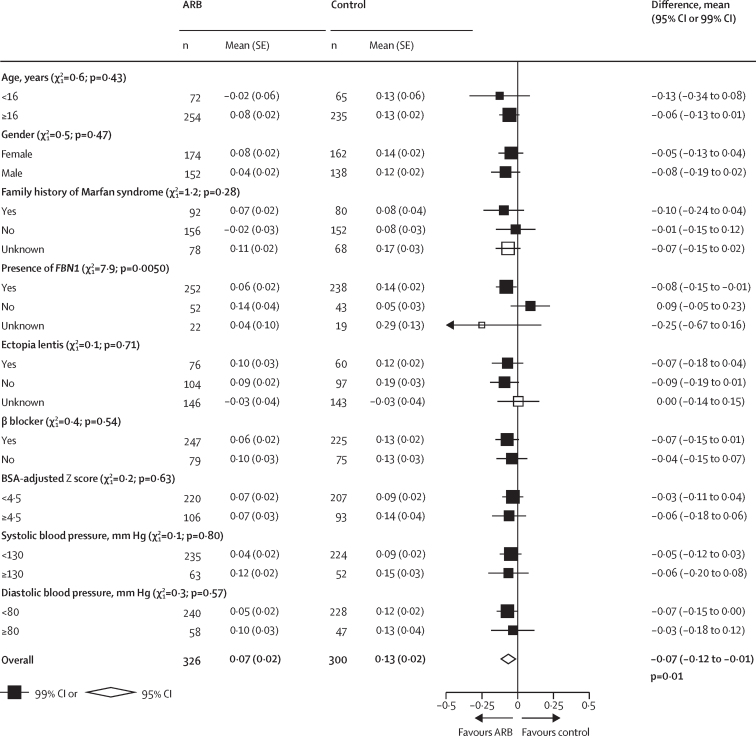
Annual rate of change of BSA-adjusted aortic root dimension Z score at the sinuses of Valsalva, by subgroups (ARB *vs* control) ARB=angiotensin receptor blocker. BSA=body surface area.

Although there was no evidence of heterogeneity between the overall results of the four contributing trials (heterogeneity p=0·11; figure 1), within these trials, there was significant heterogeneity of treatment effects with an ARB between the 490 participants with a documented pathogenic variant in *FBN1*, compared with the 95 participants who did not have a pathogenic variant in *FBN1* (figure 2; appendix p 8; heterogeneity p=0·0050). There was no significant heterogeneity of the effects of an ARB in any of the other prespecified subgroups. In particular, the mean annual change in aortic root Z score was similar irrespective of whether or not patients were taking a β blocker at baseline (heterogeneity p=0·54).

Secondary analyses were done to assess the sensitivity of the findings to different methods of aortic root size measurement. For the secondary outcome of absolute aortic dimension, the mean annual change was 0·38 mm (SE 0·04) in patients allocated ARB and 0·52 mm (SE 0·04) in patients allocated control, resulting in a mean difference of –0·14 mm (95% CI –0·26 to –0·02; p=0·025; appendix p 9). Findings were similar when the analysis included one confounded trial^22^ in which there were different dosing strategies for β blockers in the ARB and control groups during the study (mean difference –0·08 [95% CI –0·13 to –0·03]; p=0·0009; appendix p 10), and were also similar when the Z scores were calculated using the method described by Campens and colleagues^29^ (mean difference –0·04 [95% CI –0·07 to –0·01]; p=0·0071; appendix p 11) or by Pettersen and colleagues^30^ (mean difference –0·04 [95% CI –0·07 to –0·01]; p=0·017; appendix p 12).

**Figure 3:**
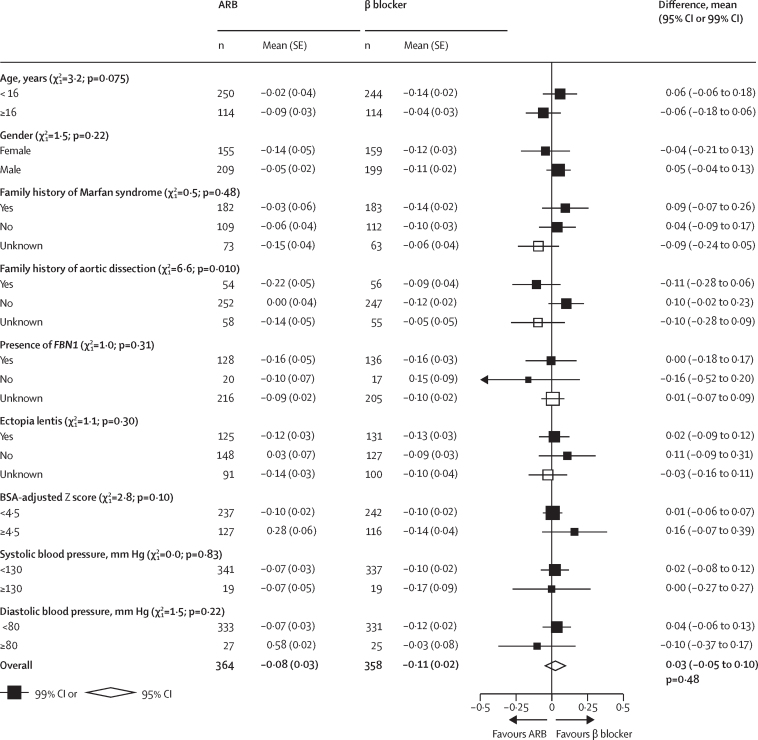
Annual rate of change of BSA-adjusted aortic root dimension Z score at the sinuses of Valsalva, by subgroups (ARB *vs* β blocker) ARB=angiotensin receptor blocker. BSA=body surface area.

Individual participant data were available from three trials of ARB versus β blockers, including 766 patients (384 assigned to ARB and 382 to β blocker).^17^, ^21^, ^24^ The mean age of participants was 14 years (SD 10); 336 (44%) of 766 were female, and 280 (86%) of 327 genotyped individuals had a pathogenic variant in *FBN1* (table 2). The baseline mean Z score was 4·18 (SD 1·71) in patients allocated to ARB and 4·03 (SD 1·50) in patients allocated to β blocker. The mean annual change of the Z score during follow-up was –0·08 (SE 0·03) in the ARB group and –0·11 (SE 0·02) in the β-blocker group, and the mean difference in the change in Z scores between ARB groups and β-blocker groups was not significant (0·03 [95% CI –0·05 to 0·10]; p=0·48]; figure 1). There were no significant differences in aortic Z score when using other methods^29^, ^30^ (appendix pp 11–12). Similarly, there were no significant differences between the two groups in other measures of change in aortic dimensions, including absolute aortic dimension (appendix p 9). There was some evidence of heterogeneity in the Z score difference between ARB and β blockers depending on family history of aortic dissection (favouring ARB in the 110 patients with such a family history, heterogeneity p=0·010), but otherwise no evidence of heterogeneity in any of the other prespecified subgroups (figure 3).

In the trials of ARB versus control, there was no significant difference in the proportion of patients who had the composite outcome of aortic dissection, aortic root surgery, or death during study follow-up (30 [8%] of 353 ARB *vs* 27 [8%] of 323 control, p=0·86). Nor was there any evidence of difference in this composite outcome in the trials of ARB versus β blockers (21 [5%] of 384 ARB *vs* 14 [4%] of 382 β blockers, p=0·23; appendix p 3).

Combining the results of the four trials of ARB versus control with the three trials of ARB versus β blockers allowed us to indirectly assess β blockers versus control. With such an analysis, the difference in mean absolute annual change in the aortic root Z score between β blocker and control was –0·09 (95% CI –0·18 to 0·00; p=0·042; ie, similar to the direct estimate when comparing ARB with control). Since the trials of ARB versus control and ARB versus β blockers included patients within different age ranges, in a post-hoc exploratory analysis we examined the indirect comparison of β blockers versus control separately in patients younger than 16 years and patients aged 16 years or older. We found no evidence that the effect of a β blocker differed in those younger than 16 years compared with those aged 16 years and older (heterogeneity p=0·09).

We also prespecified a range of other secondary analyses in our published protocol.^25^ These included assessments of aortic dimension at locations other than the sinuses of Valsalva, assessments using different imaging methods, and analyses of haemodynamic variables (eg, blood pressure) and other physical measurements. The numbers of patients available for such analyses varied, depending on what was included in each trial's case report forms, and the results are summarised in the appendix (p 4). None of these results are qualitatively inconsistent with the main findings. Additionally, we prespecified exploratory analyses using a random effects model: the absolute differences for each of these were similar to those derived from our prespecified method of analysis (a so called fixed effect analysis), but with wider CIs (as would be expected; appendix p 6). Finally, we prespecified that for baseline groups defined by age, systolic blood pressure, diastolic blood pressure, and BSA-adjusted Z score we would do additional interaction tests in which these factors were considered as continuous rather than categorical variables. For all eight interaction tests (four in each of the two groups of trials) there was no good evidence that the effect on aortic root dimension Z score varied significantly depending on the baseline characteristic (the smallest of these interaction p values was 0·05, which is not significant given the multiple tests that were done).

## Discussion

Marfan syndrome affects about one in 5000 people, which corresponds to a global total of 1·6 million people.^38^ Marfan syndrome causes a dramatically increased risk of aortic complications, mainly aortic dissection, commonly resulting in premature death or disability: a 2018 study reported 11 fatal aortic complications among 412 patients with Marfan syndrome, compared to only six such complications among 41 500 age-matched and sex-matched controls over a comparable period (hazard ratio 194·6 [95% CI 67·4–561·7]).^7^ Prophylactic surgery to replace the aortic root is recommended in cases in which a large or rapidly expanding aneurysm presents an imminent risk of aortic dissection, but such surgery is itself associated with morbidity, occasional mortality, and is not available in all health-care systems. Effective medical therapy that is well-tolerated by both children and adults could delay or prevent the need for surgery.

This meta-analysis shows that among patients with Marfan syndrome who had had no previous aortic surgery, an ARB reduces the rate of increase of the aortic root Z score by around one half and that this effect seems to be in addition to any effects of β blockers (which are discussed later). The robustness of our findings on the effects of an ARB is reinforced by several observations that, since they depended on the availability of individual participant data, went beyond the results of previous meta-analyses of tabular data.^26^, ^27^, ^28^ The first of these observations is that, although in general there was little evidence of heterogeneity of the effect of ARBs among the prespecified subgroups, ARBs had a significantly greater effect on aortic root dimension Z score among patients with a known pathogenic variant in the *FBN1* gene. Given that *FBN1* variant status is a marker of the certainty of a diagnosis of Marfan syndrome, this finding is what might be expected if an ARB is effective at slowing root expansion in this condition. The second observation was that all of the prespecified methods for estimating change in root size yielded significant results, so that our findings were not dependent on the performance of a particular method; this, again, is as might be expected if an ARB is effective.

Our analyses are most informative about the effects of an ARB, because these analyses involved meta-analyses of trials making direct comparisons of an ARB versus control. Our results are less definitive for β blockers than ARBs, because the β-blocker analyses depended on indirect comparisons of two groups of trials (four comparing an ARB *vs* control and three comparing an ARB *vs* a β blocker). Our indirect estimate of the effect of β blockers is consistent with the results of one small trial that directly compared a β blocker with no treatment.^10^ However, our estimates rely on the assumption that the effects of ARBs and β blockers are independent of each other (ie, that the effects of each drug on change in aortic Z score are additive). Our observation of no significant heterogeneity of the effect of ARBs depending on concomitant use of β blockers suggests this assumption is a reasonable one, but does not prove the assumption to be correct. Since the trials of an ARB versus control and of an ARB versus a β blocker differed substantially in average age at entry (13·9 years for ARB *vs* β blockers and 28·5 years for ARB *vs* control), we assessed the separate effects of a β blocker in those younger than 16 years and those aged 16 years and older in stratified analyses. Although we found no evidence that effects of β blockers varied significantly depending on age, there was little power to detect any true heterogenity that might have been present.

The clinical significance of our results is informed by sample size calculations done by the Pediatric Heart Network investigators^24^ who assumed that, in an adult Marfan syndrome population with a mean age of 20 years and Z score of 4·3, the threshold for aortic surgery would be reached in about 15 years (when the Z score is 7·3 and the aortic root diameter is 5·04 cm). Reducing this annual rate of change from 0·20 to 0·12 (ie, annual reduction of 0·08) would therefore increase the expected time to surgery by about 10 years (since, at an annual increase in Z score of 0·12 rather than 0·20, it would take 25 rather than 15 years for the Z score to increase from 4·3 to 7·3). Such an annual reduction is consistent with the absolute changes in Z score in our analyses (–0·07 for an ARB and –0·09 for a β blocker).

A limitation of our analysis is that, despite making every effort to obtain all available trial datasets, not all were available for individual data analysis: one published trial^16^ did not contribute data but was very small (34 participants followed up for only 6 months) and its conclusions were consistent with the results of the meta-analysis, so it would not have influenced our conclusions. Another trial, of moderate size (n=262, follow-up 48 months), was not available from the investigators.^32^ The main findings of this second trial from which data were unavailable have, however, been reported in abstract form and showed that the combination of an ARB (losartan) and a β blocker (nebivolol) reduced progression as compared with either treatment alone (p=0·009),^32^ which is consistent with the main findings of the meta-analysis. Of the trials included in our meta-analysis, one used irbesartan^23^ and losartan was used in the others, hence the amount of data available for irbesartan was scarce compared with that for losartan. No trials randomly allocated participants to prespecified ARB dosing strategies or to different agents. Consequently, it was not possible to explore whether any particular ARB selection or dosing strategy was superior to any other (and similar limitations apply to β blockers). The generalisability of our findings to older adults with Marfan syndrome is somewhat uncertain, as only 11% of the randomly assigned patients were aged 40 years or older and only 6% were aged 50 years or older. Finally, even in this meta-analysis of all eligible and available trials, the number of patients who had major clinical outcomes was too small to provide sufficient statistical power to detect benefit on such outcomes over the relatively short duration of the trials.

In summary, in these trials of patients with Marfan syndrome, ARBs reduced the rate of enlargement of the aortic root by about one half, including among those already taking a β blocker. The effect was particularly large among patients with a pathogenic *FBN1* variant, strengthening the main finding. The effects of β blockers were similar in magnitude to those of ARBs. Moreover, for ARB versus control, there was no evidence that the effect size depended on use of β blockers. Our findings therefore suggest that, if tolerated, the combination of a β blocker and ARB would reduce the rate of enlargement of the aortic root by at least one half, and potentially by much more than this which, if maintained over a sustained period, would be expected to delay the need for surgery substantially.

## Data sharing

The NIH NHLBI PHN Marfan Study dataset was used in preparation of this work. Data were downloaded from https://www.pediatricheartnetwork.org/login/ on Oct 15, 2019. Other individual participant datasets were provided to the Marfan Treatment Trialists’ (MTT) secretariat in Oxford, UK, by the trialists. Data sharing requests for these trials should be sent to the principal investigators of the trials, who are: MF (m.flather@uea.ac.uk) for the AIMS trial; GS (gsandor@telus.net) for the Canadian Trial; MG (m.groenink@amc.uva.nl) for the COMPARE trial; JDB (Julie.DeBacker@UGent.be) for the Ghent Marfan trial; GT-T (gteixido@vhebron.net) for the LOAT trial; GJ (guillaume.jondeau@aphp.fr) for the Marfan-Sartan trial; RL (ron.lacro@cardio.chboston.org) for the PHN trial; and M-HW (wumh@ntu.edu.tw) for the Taiwanese trial.

## Declaration of interests

AP has received grants paid to his institutions from the Marfan Foundation, the British Heart Foundation, the UK National Institute for Health and Care Research Biomedical Research Centre, the Gibson fund, Oxford University Hospitals Charitable Fund, and the Academy of Medical Sciences Clinical Lecturer Starter Grant scheme (which is administered by the Academy on behalf of the Academy, the Wellcome Trust, the UK Medical Research Council [MRC], the British Heart Foundation, Arthritis Research UK, Prostate Cancer UK, and the Royal College of Physicians). CR has received institutional grants from the British Heart Foundation and UK MRC. CBa has received institutional grants from the British Heart Foundation, UK MRC, NIHR, and Boehringer Ingelheim (EMPA-KIDNEY trial).
