# Supplementary material for: Angiotensin receptor blockers and β-blockers in Marfan syndrome: an individual patient data meta-analysis of randomised trials
Source: Lancet. Author manuscript; Available in PMC 2022 Sep 23. (PMC7613630; doi:10.1016/S0140-6736(22)01534-3)

Angiotensin receptor blockers and Beta-blockers in Marfan syndrome: an individual patient data meta-analysis of randomised trials

Online Supplement, Table of Contents

Supplementary tables **Page**

1. Search strategy 2
2. Effects of ARB vs control and ARB vs β-blocker on aortic complications and mortality 3
3. Effects of ARB vs control and ARB vs β-blocker on the annual rate of change in other pre-specified outcomes 4
4. Sensitivity analysis: Annual rate of change of the absolute aortic dimension at the sinuses of Valsalva by different imaging methods 5
5. Sensitivity analysis: Effect on annual rate of change in BSA−adjusted aortic root dimension Z score at the sinuses of Valsalva when analysed using standard random effects meta-analysis 6

Supplementary figures

1. PRISMA IPD flow diagram 7
2. Annual rate of change of BSA-adjusted aortic root dimension z-score at the sinuses of Valsalva in patients with confirmed FBN1 pathogenic variant 8
3. Annual rate of change of the absolute aortic dimension at the sinuses of Valsalva 9
4. Sensitivity analysis: annual rate of change of BSA−adjusted aortic root dimension z−score at the sinuses of Valsalva after including Taiwan trial 10
5. Sensitivity analysis: annual rate of change of BSA−adjusted aortic root dimension z−score at the sinuses of Valsalva estimated using the Campens method 11
6. Sensitivity analysis: annual rate of change of BSA−adjusted aortic root dimension z−score at the sinuses of Valsalva estimated using the Pettersen method 12

Webtable 1: Search Strategy

	Embase		Medline		CENTRAL
	1	Randomized controlled trial/	1	Randomized Controlled Trial/	(“Marfan”):ti,ab,kw
	2	Controlled clinical trial/	2	Controlled Clinical Trial/	
	3	random$.ti,ab.	3	Randomized.ab.	
	4	randomization/	4	placebo.ab.	
	5	intermethod comparison/	5	clinical trials as topic.sh.	
	6	placebo.ti,ab.	6	randomly.ab.	
	7	(compare or compared or comparison).ti.	7	trial.ti.	
	8	((evaluated or evaluate or evaluating or assessed or assess) and (compare or compared or comparing or comparison)).ab.	8	1 or 2 or 3 or 4 or 5 or 6 or 7	
	9	(open adj label).ti,ab.	9	exp animals/ not humans.sh.	
	10	((double or single or doubly or singly) adj (blind or blinded or blindly)).ti,ab.	10	8 not 9	
	11	double blind procedure/	11	Marfan Syndrome/ or marfan.mp.	
	12	parallel group$1.ti,ab.	12	10 and 11	
	13	(crossover or cross over).ti,ab.	13	limit 12 to rd=20201214-20211102	
	14	((assign$ or match or matched or allocation) adj5 (alternate or group$1 or intervention$1 or patient$1 or subject$1 or participant$1)).ti,ab.	14	limit 12 to dt=20201214-20211102	
	15	(assigned or allocated).ti,ab.	15	13 or 14	
	16	(controlled adj7 (study or design or trial)).ti,ab.			
	17	(volunteer or volunteers).ti,ab.			
	18	human experiment/			
	19	trial.ti.			
	20	or/1-19			
	21	(random$ adj sampl$ adj7 (cross section$ or questionnaire$1 or survey$ or database$1)).ti,ab. not (comparative study/ or controlled study/ or randomi?ed controlled.ti,ab. or randomly assigned.ti,ab.)			
	22	Cross-sectional study/ not (randomized controlled trial/ or controlled clinical study/ or controlled study/ or randomi?ed controlled.ti,ab. or control group$1.ti,ab.)			
	23	(((case adj control$) and random$) not randomi?ed controlled).ti,ab.			
	24	(Systematic review not (trial or study)).ti.			
	25	(nonrandom$ not random$).ti,ab.			
	26	Random field$.ti,ab.			
	27	(random cluster adj3 sampl$).ti,ab.			
	28	(review.ab. and review.pt.) not trial.ti.			
	29	we searched.ab. and (review.ti. or review.pt.)			
	30	update review.ab.			
	31	(databases adj4 searched).ab.			
	32	(rat or rats or mouse or mice or swine or porcine or murine or sheep or lambs or pigs or piglets or rabbit or rabbits or cat or cats or dog or dogs or cattle or bovine or monkey or monkeys or trout or marmoset$1).ti. and animal experiment/			
	33	Animal experiment/ not (human experiment/ or human/)			
	34	or/21-33			
	35	20 not 34			
	36	Marfan syndrome/ or Marfan.mp.			
	37	35 and 36			
**Sources**	Ovid SP was used to search Embase and Medline for relevant trial publications.The latest filter is found in the Cochrane Handbook for Systematic Reviews of Interventions: 4.S1 Technical Supplement to Chapter 4: Searching for and selecting studies | Cochrane Training Medline filter is found in Box 3.d. Embase filter is found in Box 3.e. Cochrane CENTRAL registry https://www.cochranelibrary.com/central. There were no language restrictions.				
**Search period**	Embase 1974 to 2nd November 2021. Medline (Ovid MEDLINE® Epub Ahead of Print, In-Process & Other Non-Indexed Citations, Ovid MEDLINE® Daily and Ovid MEDLINE®) 1946 to 2nd November 2021. All CENTRAL records to 2nd November 2021				
**Citation processing**	LH and HH completed the search. Results were downloaded into EndNote X7 where duplicates were removed. Reasons for exclusion were recorded in custom fields. The review of search results was done independently by two authors (two of HH, KW, KD and LH) and any adjudication was carried out by AP.				

Webtable 2: Effects of ARB vs control and ARB vs β-blocker on aortic complications and mortality

	ARB vs Control		ARB vs β-blocker	
	**ARB (*n*=353)**	**Control (*n*=323)**	**ARB (*n*=384)**	**β -blocker (*n*=382)**
Aortic dissection	0 (0%)	2 (2%)	2 (1%)	3 (1%)
Aortic surgery	30 (8%)	23 (7%)	19 (5%)	12 (3%)
Death	0 (0%)	3 (1%)	2 (1%)	1 (<0.5%)
Any of the above	30 (8%)	27 (8%)	21 (5%)	14 (4%)

Results are count (%).

Webtable 3: Effects of ARB vs control and ARB vs β-blocker on the annual rate of change in other pre-specified outcomes

Outcome	'Active' vs 'comparator'				
	ARB vs control (direct comparison)		ARB vs β-blocker (direct comparison)		β-blocker vs control (indirect comparison)
	n	Mean difference (95% CI)	*n*	Mean difference (95% CI)	Mean difference (95% CI)
Absolute aortic dimensions at the ascending aorta (mm)	575	0.00 (-0.12 to 0.13)	656	-0.01 (-0.15 to 0.13)	0.01 (-0.18 to 0.20)
Absolute aortic dimensions at the aortic annulus (mm)	294	0.04 (-0.26 to 0.35)	593	0.16 (0.00 to 0.32)	-0.11 (-0.46 to 0.23)
Absolute aortic dimensions at the sino-tubular junction (mm)	454	0.10 (-0.13 to 0.33)	556	0.27 (0.02 to 0.52)	-0.17 (-0.51 to 0.17)
Absolute aortic dimensions at the aortic arch (mm)*	542	-0.03 (-0.17 to 0.10)	17	0.95 (-1.74 to 3.63)	-0.98 (-3.67 to 1.71)
Absolute aortic dimensions at the descending aorta (mm)†	517	-0.03 (-0.16 to 0.10)	577	-0.16 (-0.35 to 0.02)	0.13 (-0.10 to 0.36)
Absolute dimension of pulmonary artery (mm)†‡	64	-0.36 (-2.11 to 1.39)	493	0.02 (-0.27 to 0.31)	-0.38 (-2.16 to 1.40)
Systolic blood pressure (mm Hg)	573	-1.54 (-2.43 to -0.65)	743	-0.44 (-1.18 to 0.30)	-1.10 (-2.26 to 0.05)
Diastolic blood pressure (mm Hg)	572	-1.08 (-1.73 to -0.42)	743	0.43 (-0.25 to 1.10)	-1.50 (-2.44 to -0.56)
Heart rate (beats/minute)	432	0.21 (-0.85 to 1.27)	722	2.20 (1.21 to 3.19)	-1.99 (-3.44 to -0.53)
Pulse pressure (mm Hg)	572	- 0.33 (-1.06 to 0.39)	743	-0.73 (-1.22 to -0.25)	0.40 (-0.48 to 1.27)
Mean arterial pressure (mm Hg)	572	-1.26 (-1.92 to -0.60)	743	0.10 (-0.56 to 0.75)	-1.36 (-2.28 to -0.43)
Height (cm)	484	0.03 (-0.36 to 0.43)	744	0.10 (-0.40 to 0.59)	-0.06 (-0.69 to 0.57)
Weight (kg)	485	0.20 (-0.31 to 0.71)	744	0.00 (-0.39 to 0.39)	0.20 (-0.44 to 0.84)
Body Surface Area, m^2	484	0.00 (-0.01 to 0.01)	744	0.00 (-0.01 to 0.01)	0.00 (-0.01 to 0.01)
BMI, kg/m^2	484	0.04 (-0.09 to 0.17)	744	0.00 (-0.11 to 0.11)	0.04 (-0.13 to 0.20)
Arm span (cm) †¶	83	-0.21 (-1.57 to 1.16)	589	0.07 (-0.55 to 0.70)	-0.28 (-1.78 to 1.22)
Upper segment measurement (cm) †¶	84	-1.35 (-3.13 to 0.43)	587	-0.19 (-0.55 to 0.18)	-1.16 (-2.98 to 0.66)
Lower segment measurement (cm) †¶	84	1.29 (-0.19 to 2.78)	587	0.17 (-0.29 to 0.62)	1.13 (-0.43 to 2.68)

For each comparison positive values of the difference reflect a higher mean value in the 'active' arm than in the 'comparator' arm

* Canada only, for ARB vs β-blocker.
† PHN only, for ARB vs β-blocker.
‡ Marfan-Sartan only, for ARB vs control.
¶ AIMS only, for ARB vs control

Webtable 4: Sensitivity analysis: Annual rate of change of the absolute aortic dimension at the sinuses of Valsalva by different imaging methods

Trial	Imaging method	Active		Control		Mean difference (95% CI)
		n	Mean (95% CI)	*n*	Mean (95% CI)	
AIMS	Echo systole (primary method)	95	0.57 (0.38 to 0.75)	85	0.83 (0.65 to 1.01)	-0.26 (-0.52 to -0.01)
	Echo diastole (alternative method)	95	0.65 (0.46 to 0.84)	85	0.89 (0.73 to 1.05)	-0.24 (-0.49 to 0.01)
PHN	Echo systole (primary method)	299	0.93 (0.70 to 1.15)	294	0.75 (0.63 to 0.86)	0.18 (-0.07 to 0.43)
	Echo diastole (alternative method)	296	0.85 (0.63 to 1.08)	290	0.63 (0.52 to 0.73)	0.23 (-0.02 to 0.48)
LOAT*	MRI diastole (primary method)	57	0.37 (0.19 to 0.55)	55	0.47 (0.31 to 0.64)	-0.10 (-0.34 to 0.14)
	Echo diastole (alternative method)	64	0.68 (0.47 to 0.89)	64	0.47 (0.32 to 0.62)	0.21 (-0.05 to 0.47)

* MRI measurements recorded at baseline and month 36 only. Echo measurements recorded at baseline and months 12, 24 and 36

Webtable 5: Sensitivity analysis: Effect on annual rate of change in BSA−adjusted aortic root dimension Z score at the sinuses of Valsalva when analysed using standard random effects meta-analysis

Comparison	Mean difference in annual rate of change in BSA−adjusted aortic root dimension Z score (95% CI)	p-value
ARB vs control (direct comparison)	-0.06 (-0.15 to 0.03)	0.17
ARB vs β-blocker (direct comparison)	0.02 (-0.14 to 0.19)	0.80
β-blocker vs control (indirect comparison)	-0.08 (-0.27 to 0.10)	0.39

For each comparison positive values of the difference reflect a higher mean value in the ‘active’ arm than in the ‘comparator’ arm. The random-effects analysis (which assumes that the trials are ‘representative’ of an underlying population of trials) estimates both the average and variability of effects across studies and incorporates both into the effect estimates shown. The pre-specified primary analysis shown in the main results was the assumption-free inverse-variance-weighted average of the trial results, which makes no assumptions about the true heterogeneity of effects between studies (in particular it does not assume that such heterogeneity is zero) and should be used to guide clinical interpretation of the results.

Webfigure 1: PRISMA IPD flow diagram

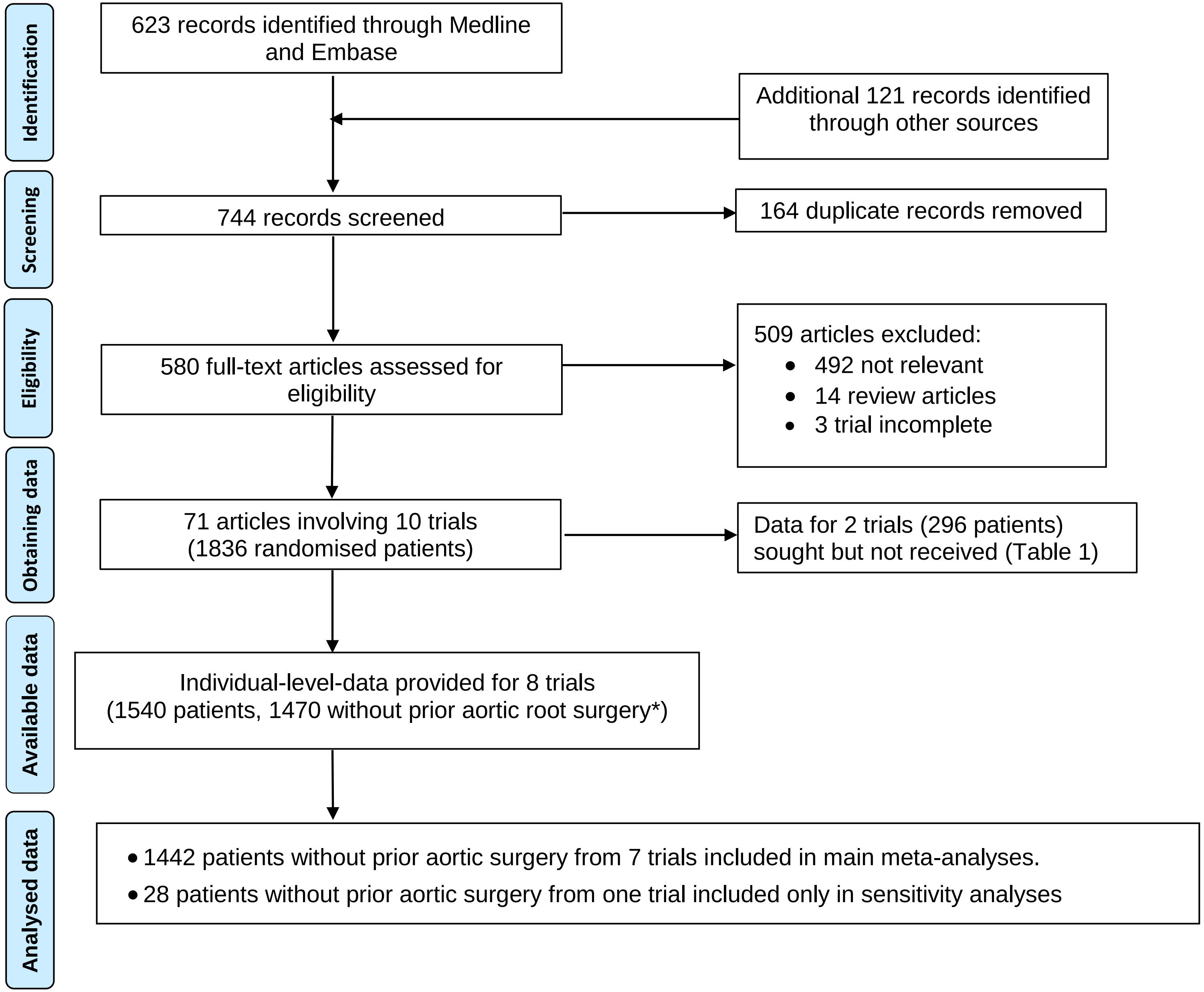

*70 patients with prior aortic root surgery at enrolment are excluded: 2 (ARB) vs 5 (placebo) from Ghent Marfan and 27 (ARB) vs 36 (control) patients from COMPARE.

Webfigure 2: Annual rate of change of BSA−adjusted aortic root dimension z−score at the sinuses of Valsalva in patients with confirmed FBN1 pathogenic variant

Trial	Annual change: ARB n	Annual change: ARB mean (SE)	Annual change: Comparator arm n	Annual change: Comparator arm mean (SE)	Difference mean (95% or 99% CI)
**ARB vs Control (χ^2_3= 5.0; p=0.17)**					
Marfan−Sartan	114	−0.03 (0.04)	112	−0.03 (0.05)	0.00 (−0.17, 0.17)
COMPARE	59	0.09 (0.02)	59	0.18 (0.03)	−0.09 (−0.18, 0.00)
AIMS	69	0.02 (0.06)	62	0.18 (0.04)	−0.15 (−0.32, 0.02)
Ghent Marfan	10	0.32 (0.24)	5	0.02 (0.08)	0.30 (−0.35, 0.94)
**Overall**	**252**	**0.06 (0.02)**	**238**	**0.14 (0.02)**	**−0.08 (−0.13, −0.03) p=0.0041**
**ARB vs Beta−blocker (χ^2_2= 3.6; p=0.06)**					
PHN	87	0.02 (0.12)	89	−0.17 (0.03)	0.19 (−0.13, 0.50)
LOAT	41	−0.20 (0.06)	42	−0.10 (0.06)	−0.10 (−0.31, 0.12)
**Overall**	**128**	**−0.16 (0.05)**	**136**	**−0.16 (0.03)**	**0.00 (−0.14, 0.13) p=0.95**

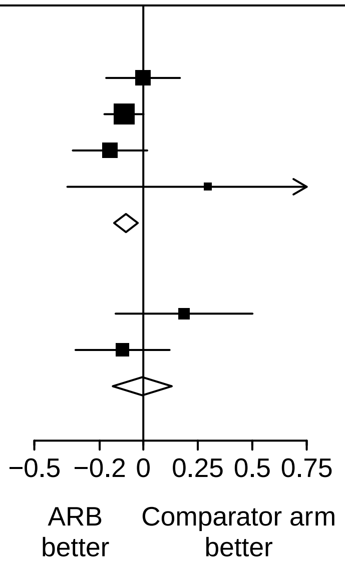

■ 99% or ◇ 95% CI

Webfigure 3: Annual rate of change of the absolute aortic dimension at the sinuses of Valsalva

Trial	ARB n	ARB mean (SE)	Comparator arm n	Comparator arm mean (SE)	Difference, mm mean (95% or 99% CI)
	Annual change, mm				
**ARB vs Control (χ^2_3= 4.5; p=0.21)**					
Marfan−Sartan	145	0.42 (0.07)	145	0.42 (0.09)	0.00 (−0.29, 0.29)
COMPARE	77	0.28 (0.06)	67	0.45 (0.07)	−0.18 (−0.40, 0.05)
AIMS	95	0.57 (0.09)	85	0.83 (0.09)	−0.26 (−0.60, 0.07)
Ghent Marfan	10	0.89 (0.57)	5	0.18 (0.20)	0.71 (−0.85, 2.27)
**Overall**	**327**	**0.38 (0.04)**	**302**	**0.52 (0.04)**	**−0.14 (−0.26, −0.02) p=0.03**
**ARB vs Beta−blocker (χ^2_2= 5.1; p=0.08)**					
PHN	299	0.93 (0.11)	294	0.75 (0.06)	0.18 (−0.15, 0.51)
LOAT	57	0.37 (0.09)	55	0.47 (0.08)	−0.10 (−0.42, 0.22)
Canada	8	1.61 (0.64)	9	0.26 (0.54)	1.36 (−0.78, 3.50)
**Overall**	**364**	**0.60 (0.07)**	**358**	**0.66 (0.05)**	**0.05 (−0.12, 0.22) p=0.57**

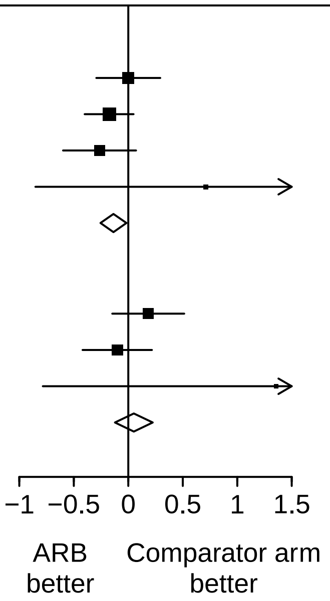

99% or 95% CI

Webfigure 4: Sensitivity analysis: annual rate of change of BSA−adjusted aortic root dimension z−score at the sinuses of Valsalva after including Taiwan trial

Trial	Annual change: ARB n	Annual change: ARB mean (SE)	Annual change: Control n	Annual change: Control mean (SE)	Difference mean (95% or 99% CI)
**ARB vs Control (χ^2_3= 12.5; p=0.01)**					
Marfan−Sartan	145	−0.03 (0.04)	143	−0.03 (0.04)	0.00 (−0.14, 0.15)
COMPARE	77	0.11 (0.02)	67	0.17 (0.03)	−0.07 (−0.15, 0.02)
AIMS	94	0.03 (0.05)	85	0.19 (0.04)	−0.16 (−0.32, 0.00)
Ghent Marfan	10	0.32 (0.24)	5	0.02 (0.08)	0.30 (−0.35, 0.94)
Taiwan	15	−0.02 (0.05)	13	0.28 (0.07)	−0.31 (−0.54, −0.07)
**Overall**	**341**	**0.06 (0.02)**	**313**	**0.14 (0.02)**	**−0.08 (−0.13, −0.03) p=0.0009**

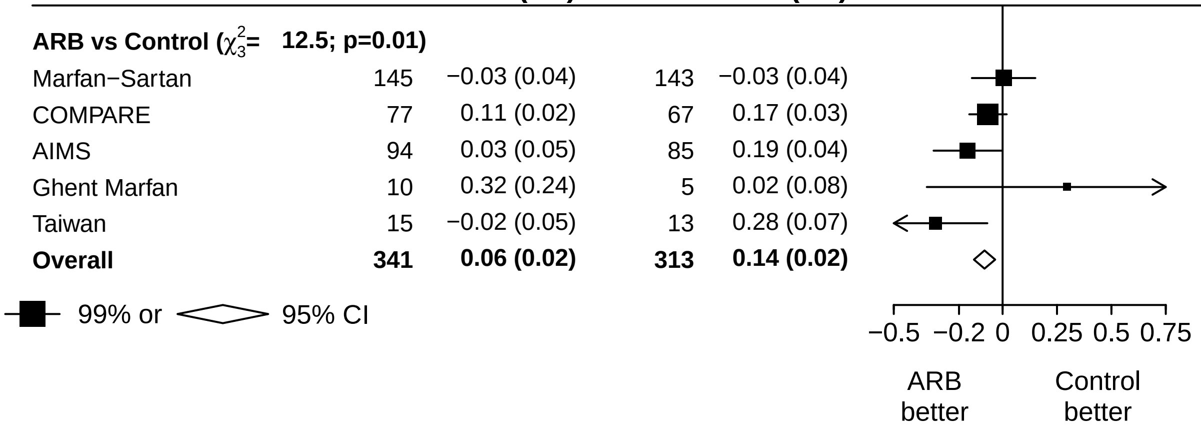

Webfigure 5: Sensitivity analysis: annual rate of change of BSA−adjusted aortic root dimension z−score at the sinuses of Valsalva estimated using the Campens method

Trial	ARB n	ARB mean (SE)	Comparator arm n	Comparator arm mean (SE)	Difference mean (95% or 99% CI)
**ARB vs Control (χ^2_3= 5.1; p=0.16)**					
Marfan−Sartan	145	0.00 (0.02)	143	0.01 (0.02)	−0.01 (−0.08, 0.06)
COMPARE	77	0.03 (0.01)	67	0.07 (0.02)	−0.04 (−0.10, 0.01)
AIMS	94	−0.02 (0.03)	85	0.06 (0.02)	−0.08 (−0.17, 0.00)
Ghent Marfan	10	0.21 (0.18)	5	−0.02 (0.06)	0.23 (−0.25, 0.71)
**Overall**	**326**	**0.01 (0.01)**	**300**	**0.05 (0.01)**	**−0.04 (−0.07, −0.01) p=0.0071**
**ARB vs Beta−blocker (χ^2_2= 8.1; p=0.02)**					
PHN	299	−0.05 (0.02)	294	−0.10 (0.01)	0.05 (−0.02, 0.12)
LOAT	57	−0.04 (0.02)	55	0.01 (0.02)	−0.05 (−0.12, 0.02)
Canada	8	0.20 (0.17)	9	−0.08 (0.16)	0.28 (−0.31, 0.87)
**Overall**	**364**	**−0.04 (0.01)**	**358**	**−0.06 (0.01)**	**0.00 (−0.04, 0.04) p=0.99**

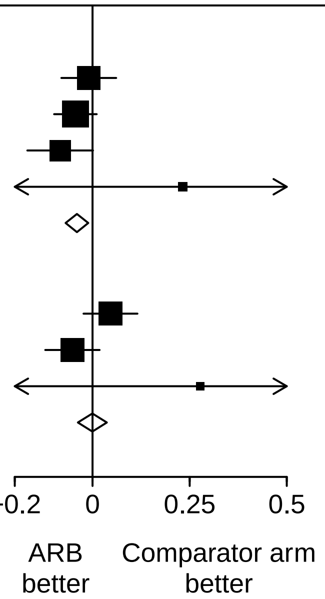

99% or 95% CI

Webfigure 6: Sensitivity analysis: annual rate of change of BSA−adjusted aortic root dimension z−score at the sinuses of Valsalva estimated using the Pettersen method

Trial	ARB n	ARB mean (SE)	Comparator arm n	Comparator arm mean (SE)	Difference mean (95% or 99% CI)
**ARB vs Control (χ^2_3= 4.7; p=0.19)**					
Marfan−Sartan	145	−0.02 (0.02)	143	−0.01 (0.03)	−0.01 (−0.09, 0.08)
COMPARE	77	0.06 (0.01)	67	0.10 (0.01)	−0.04 (−0.08, 0.01)
AIMS	94	−0.08 (0.04)	85	0.02 (0.02)	−0.10 (−0.22, 0.02)
Ghent Marfan	10	0.22 (0.17)	5	−0.01 (0.08)	0.24 (−0.26, 0.73)
**Overall**	**326**	**0.03 (0.01)**	**300**	**0.06 (0.01)**	**−0.04 (−0.07, −0.01) p=0.02**
**ARB vs Beta−blocker (χ^2_2= 3.4; p=0.18)**					
PHN	299	−0.06 (0.02)	294	−0.11 (0.02)	0.05 (−0.02, 0.12)
LOAT	57	−0.03 (0.02)	55	0.01 (0.03)	−0.04 (−0.14, 0.07)
Canada	8	0.03 (0.17)	9	−0.03 (0.14)	0.06 (−0.50, 0.62)
**Overall**	**364**	**−0.04 (0.01)**	**358**	**−0.09 (0.01)**	**0.02 (−0.02, 0.07) p=0.26**

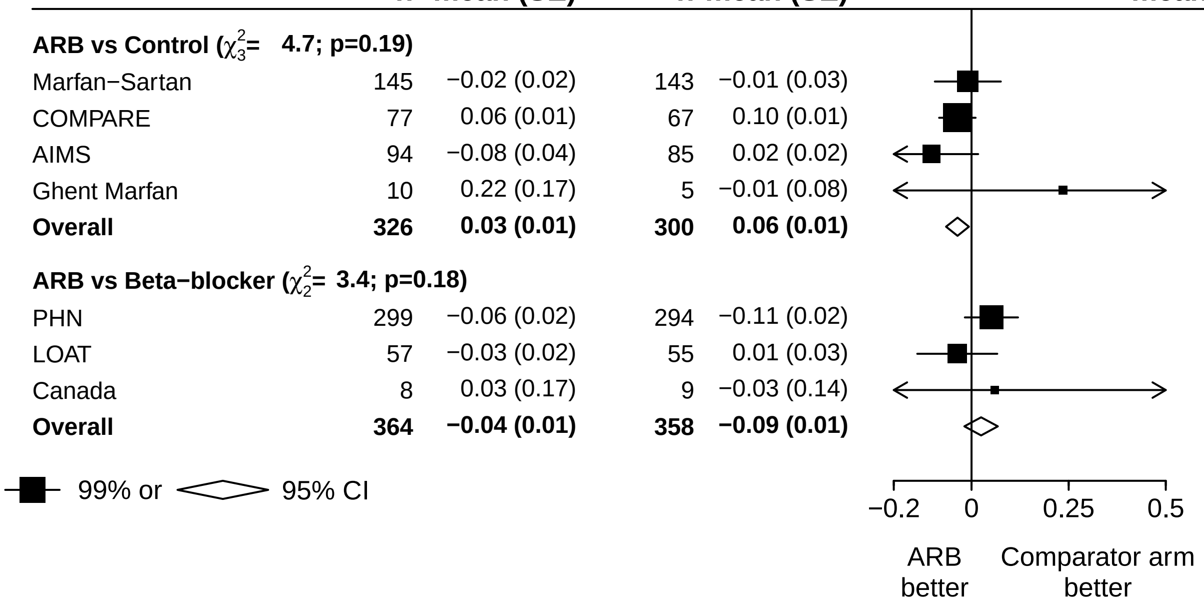

Supplement: Supplementary file 1 [file EMS152102-supplement-Appendix.pdf]
